# HERV Envelope Proteins: Physiological Role and Pathogenic Potential in Cancer and Autoimmunity

**DOI:** 10.3389/fmicb.2018.00462

**Published:** 2018-03-14

**Authors:** Nicole Grandi, Enzo Tramontano

**Affiliations:** ^1^Laboratory of Molecular Virology, Department of Life and Environmental Sciences, University of Cagliari, Cagliari, Italy; ^2^Istituto di Ricerca Genetica e Biomedica, Consiglio Nazionale delle Ricerche, Cagliari, Italy

**Keywords:** HERV, endogenous retroviruses, Env, cancer, autoimmunity, multiple sclerosis, syncytin

## Abstract

Human endogenous retroviruses (HERVs) are relics of ancient infections accounting for about the 8% of our genome. Despite their persistence in human DNA led to the accumulation of mutations, HERVs are still contributing to the human transcriptome, and a growing number of findings suggests that their expression products may have a role in various diseases. Among HERV products, the envelope proteins (Env) are currently highly investigated for their pathogenic properties, which could likely be participating to several disorders with complex etiology, particularly in the contexts of autoimmunity and cancer. In fact, HERV Env proteins have been shown, on the one side, to trigger both innate and adaptive immunity, prompting inflammatory, cytotoxic and apoptotic reactions; and, on the other side, to prevent the immune response activation, presenting immunosuppressive properties and acting as immune downregulators. In addition, HERV Env proteins have been shown to induce abnormal cell-cell fusion, possibly contributing to tumor development and metastasizing processes. Remarkably, even highly defective HERV *env* genes and alternative *env* splicing variants can provide further mechanisms of pathogenesis. A well-known example is the HERV-K(HML2) *env* gene that, depending on the presence or the absence of a 292-bp deletion, can originate two proteins of different length (Np9 and Rec) proposed to have oncogenic properties. The understanding of their involvement in complex pathological disorders made HERV Env proteins potential targets for therapeutic interventions. Of note, a monoclonal antibody directed against a HERV-W Env is currently under clinical trial as therapeutic approach for multiple sclerosis, representing the first HERV-based treatment. The present review will focus on the current knowledge of the HERV Env expression, summarizing its role in human physiology and its possible pathogenic effects in various cancer and autoimmune disorders. It moreover analyzes HERV Env possible exploitation for the development of innovative therapeutic strategies.

## Introduction

Human endogenous retroviruses (HERVs) are transposable elements acquired along primate evolution through multiple infections by now extinct exogenous retroviruses. Common to modern retroviruses, the ancient RNA genome had been reverse transcribed into a double-stranded DNA provirus and stably integrated in the host's chromosomes. In the case of HERVs, however, such ancestral infections peculiarly affected the germ line, allowing the Mendelian inheritance of HERV proviruses through the offspring. HERVs became stable components of the human genome, constituting approximately the 8% of our DNA (Lander et al., 2001). While HERV characterization at the genomic level is still ongoing, the widely reported HERV expression across tissues stimulated the search of a role in human pathogenesis. In general, however, no definitive link of any HERV sequence (and its expressed products) to human diseases has been demonstrated yet, due to a series of confounding factors that include the lack of characterization of individual HERV loci, the poor knowledge of their specific expression in healthy and diseased conditions and the absence of confirmed molecular mechanisms of pathogenesis (Grandi and Tramontano, 2017). Subsequently, even if the contribution of HERV expression to our transcriptome is by now undeniable, its significance for the tentative association with human pathogenesis has often lacked sufficient support, ending in over-interpreted conclusions (Voisset et al., 2008; Grandi and Tramontano, 2017). Far from meaning that HERV RNAs must be translated to have an effect, the uncertainty in the field demands for standardized methodologies and reliable genomic backgrounds to definitely assess which expressed HERV loci constitute a “physiological” phenomenon and which, instead, could actually have some pathological potential. In contrast, HERV protein expression, especially if exclusive of diseased tissues, is less common than RNA production and could more likely have some effects on the host, even if not intrinsically taking part to pathogenesis. An important issue to be addresses is if the specificity of expression from a given locus can be associated with a defined molecular mechanism of pathogenesis. Such knowledge could lead to the identification of individual HERV proteins involved in disease development, and thus exploitable as therapeutic targets.

The great majority of studies investigating the pathogenic role of HERV products has been focused on HERV envelope proteins (Env). In the present review, we will describe HERV-derived Env contribution to human physiology and analyze their possible impact on pathogenesis. Overall, even if not fully conclusive, the soundest evidence has been reached in cancer and autoimmunity. The main molecular mechanisms of HERV Env pathogenesis and their possible exploitation as therapeutic targets are discussed.

## Human endogenous retroviruses

**Figure 1:**
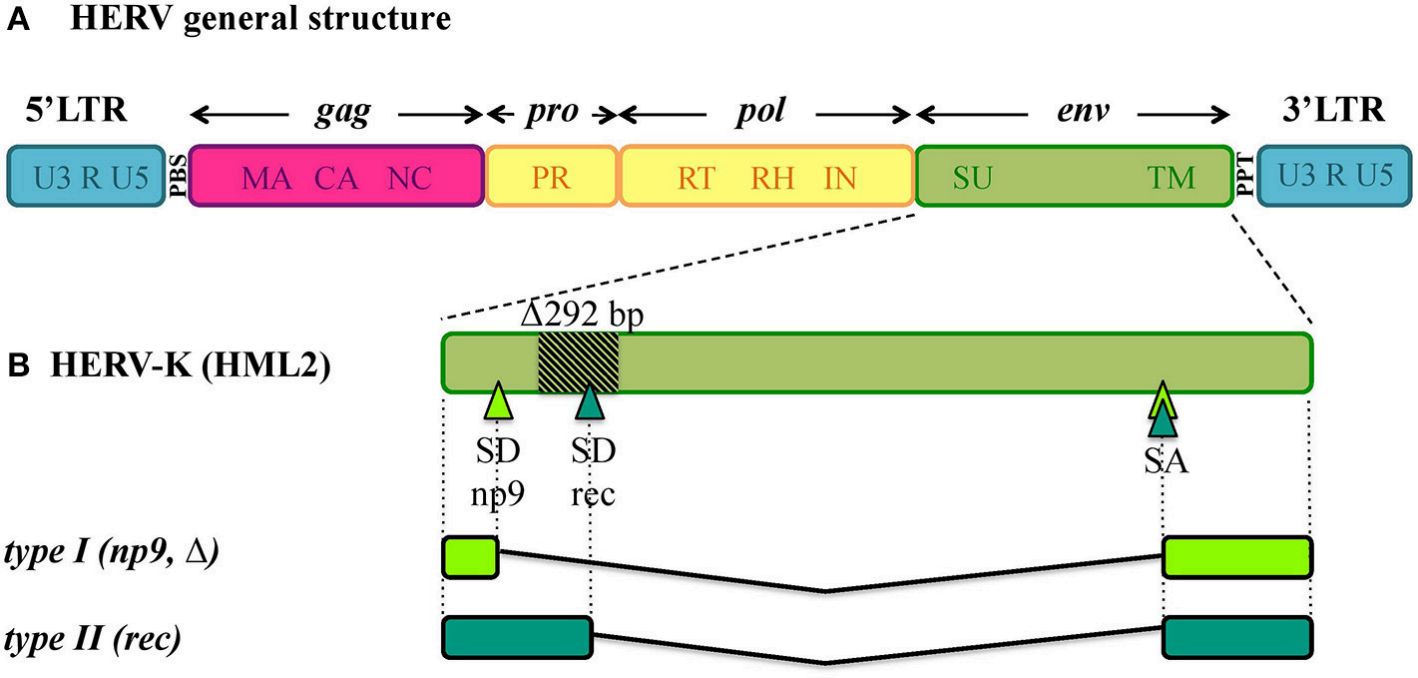
General structure of HERV DNA sequences. **(A)** *Typical structure of HERV proviruses*. The general structure of a full-length HERV proviral sequence is depicted: two Long Terminal Repeats (LTRs) flank *gag, pro, pol*, and *env* genes. The viral genes and the correspondent protein products are indicated: *gag* matrix (MA), capsid (CA) and nucleocapsid (NC); *pro-pol* protease (PR) – reverse transcriptase (RT), ribonuclease H (RH) and integrase (IN); *env* surface (SU) and transmembrane (TM). The primer binding site (PBS) and polypurine tract (PPT) are located between 5′LTR and *gag* and between *env* and 3′LTR, respectively. It is worth noting that the action of cellular editing systems and the persistence within the host genome led to the accumulation of substitutions, deletion and insertions that modified the structure of the majority of HERV proviruses, leading to coding-defective sequences and solitary LTRs formation. **(B)** *HERV-K(HML2) accessory genes*. Differently from other HERV groups, the HERV-K(HML2) *env* gene presents alternative splicing sites, leading to the presence of a full length transcript with structural significance plus two types of accessory variants, named *rec* and *np9*, that subdivide the HML2 sequences into two types. Type I HML2 elements present a characteristic 292-bp *env* deletion, leading to the use of an upstream splice donor and the subsequent production of a shorter protein, named Np9; while type II HML2 sequences retained such portion and use the downstream splice donor site to encode for a longer protein, named Rec.

Being remnants of ancient retroviral infections, HERVs show a typical proviral structure (Figure 1A), even if the action of cellular editing systems and the prolonged exposition of proviruses to the host genome substitution rate often made them coding-defective. In structurally complete HERVs, two long terminal repeats (LTRs) flank the proviral internal portion, constituted by the viral genes *gag, pro, pol* and *env*. Briefly, *gag* encodes the structural components of matrix, capsid and nucleocapsid; *pro* and *pol* specify the enzymes protease (PR), reverse transcriptase (RT) and integrase (IN); while *env* encodes Env surface (SU) and transmembrane (TM) subunits. The LTRs are formed during reverse transcription and have important regulatory functions for viral expression. HERV proviruses include moreover a primer binding site (PBS), between 5'LTR and *gag*, and a polypurine tract (PPT), between *env* and 3'LTR: the former acts as binding site for the cellular tRNA priming the (−)strand DNA synthesis, the latter serves as a primer for the (+)strand DNA synthesis. In addition, the HERV-K(HML2) group presents two accessory proteins, namely Np9 and Rec, originated by the use of alternative splicing sites during *env* transcription (Figure 1B) (Löwer et al., 1995; Armbruester et al., 2002). In fact, besides the full-length *env* mRNA, a sub-spliced *rec* mRNA can be generated by type II HML2 proviruses depending on a splicing donor site that can be lost due to a recurrent 292-bp deletion (Figure 1B). The loss of this portion characterizes type I HML2 sequences, in which an alternative splice donor site upstream of the deletion generates the *np9* mRNA (Figure 1B). As described below, both Rec and Np9 have been intensively studied for their possible role in human health. Interestingly, a recent study reported the presence of a *rec* Open Reading Frame (ORF) also in type II HERV-K(HML10) sequences, opening new perspectives for the group's possible impact on human biology (Grandi et al., 2017).

**Figure 2:**
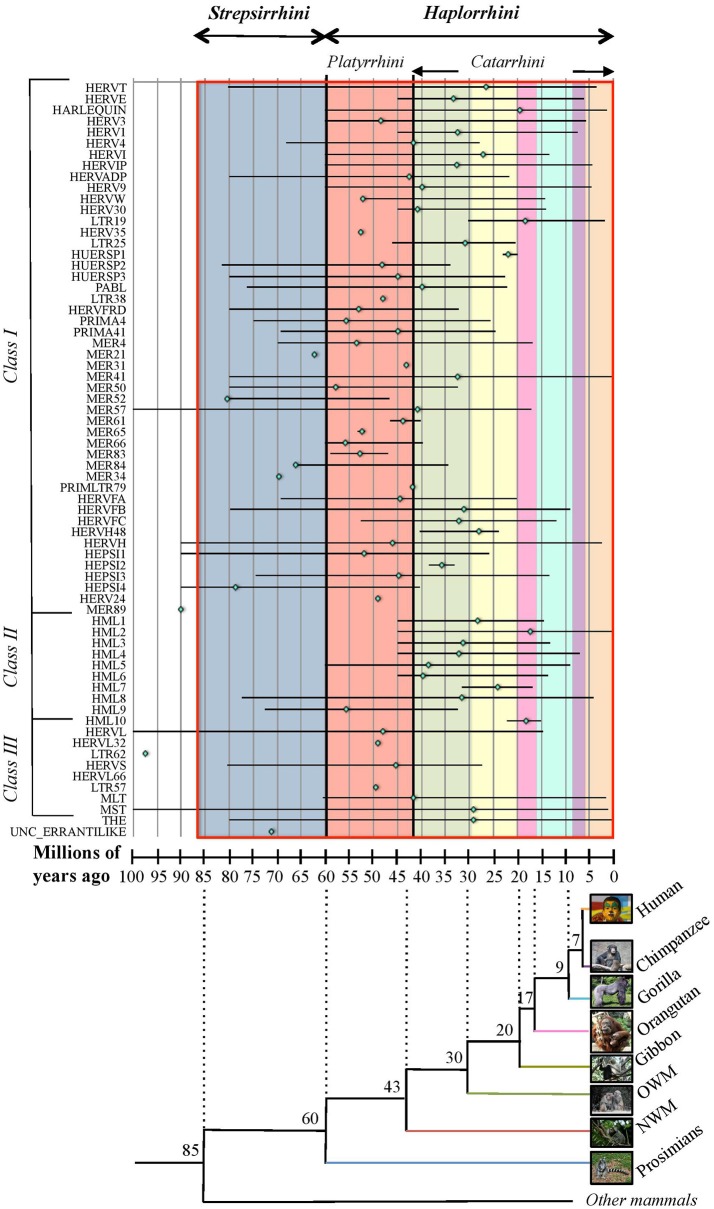
Overview of HERV groups' period of acquisition by primate lineages. For each HERV group (as listed on the x-axis), colored rhomboids indicate the average time of integration, while the global period of diffusion is delimited with a line (in millions of years, y-axis). Values were extracted from Vargiu et al. (2016) and derive from the nucleotide divergence calculated between the two LTRs of individual proviral members considering a genomic rate of 0.2% substitutions/nucleotide/millions of years. The estimated period in which the different primate species evolutionary diverged (as derived from Steiper and Young, 2006; Perelman et al., 2011) is indicated in the underlying tree at each node of separation, and is depicted in the graph by colored blocks corresponding to the first primate species infected by a certain HERV group (blue: prosimians, red: New World monkeys, green: Old World monkeys, yellow: gibbon, pink: orangutan, turquoise: gorilla, violet: chimpanzee, orange: humans). Primate parvorders (bold italic) and suborders (italic) are reported in the top of the graph, and a red line delimits the global time period of evolution of the whole order. Photo credits: human by Mostafameraji, https://commons.wikimedia.org/w/index.php?curid=61340991; chimpanzee by Thomas Lersch, https://commons.wikimedia.org/w/index.php?curid=1001910; gorilla by Adrian Pingstone, https://commons.wikimedia.org/w/index.php?curid=519340; orangutan by julubecka, https://commons.wikimedia.org/w/index.php?curid=53997414; gibbon by Raul654, https://commons.wikimedia.org/w/index.php?curid=529865; rhesus by Ltshears, https://commons.wikimedia.org/w/index.php?curid=10846501; rhesus (OWM) by Dr. Raju Kasambe, https://commons.wikimedia.org/w/index.php?curid=64614226; marmoset (NWM) by Georges Néron, https://commons.wikimedia.org/w/index.php?curid=5094199; prosimians by Sannse, https://commons.wikimedia.org/w/index.php?curid=112516.

Due to the lack of a proper nomenclature, the classification of HERVs has been for a long time incomplete and sometimes controversial. HERVs have been broadly divided in three main classes based on their similarity to exogenous retroviruses: class I (*Gammaretrovirus*- and *Epsilonretrovirus*-like), class II (*Betaretrovirus*-like) and class III (*Spumaretrovirus*-like). The individual HERV groups have been albeit designated based on discordant criteria, generating some confusion. Many HERV groups were in fact named according to the cellular tRNA recognized by their PBS (e.g. HERV-K for lysine, HERV-H for histidine), even if the subsequent characterization revealed the occurrence of PBS variants recognizing alternative tRNAs (Jern et al., 2005; Grandi et al., 2016; Vargiu et al., 2016). Other HERVs have been termed based on the name of a neighbor gene (HERV-ADP) or a particular motif (HERV-FRD). All these nomenclatures are now considered inadequate due to their poor taxonomic value, and HERV classification is based on phylogenetic relationships among the different groups. Moreover, some structural features shared by all the HERVs belonging to the same genus or class are a valuable support in better understanding retroviral phylogeny (Jern et al., 2005). According to the most updated and comprehensive analysis, performed with the software RetroTector (Sperber et al., 2007), HERVs can be classified in 39 “canonical” groups and 31 “non-canonical” clades characterized by several degrees of mosaicism (Vargiu et al., 2016) (Table 1). This classification is based on a multi-step approach and provided a remarkable background for the characterization of single HERV groups, revealing insights on HERV mosaic forms arisen from recombination and secondary integrations (Vargiu et al., 2016). It also highlighted frequent recombination events between the *env* genes of different HERV groups (“*env* snatching”), providing some positive effect on viral fitness that could be associated with both loss of *env*, favoring intragenomic spread instead of extracellular replication, and *env* acquisition, conferring a different/wider tropism (Vargiu et al., 2016). Finally, the calculation of the nucleotide divergence between proviral LTRs gave a remarkably complete (even if approximate) overview of the different HERV groups' time of integration, showing that most of them were acquired by primates from 60 to 20 million years ago (Vargiu et al., 2016) (Figure 2). Thus, the majority of HERV groups were acquired by *Haplorrhini* primates after their evolutionary separation from the elder *Strepsirrhini* suborder, being broadly divided based on their presence in both *Platyrrhini* and *Catarrhini* or in *Catarrhini* species only, due to an integration occurred after the evolutionary separation of these parvorders (~43 million years ago) (Figure 2).

**Table 1:** Identification and classification of ~3,200 HERV sequences in GRCh37/hg19 genome assembly by RetroTector (adapted from Vargiu et al., 2016).

**Class**	**Genus**	**Ex. species^a^**	**Sequences**	**Clades**	**Supergroups**	**Canonical groups**
Class I	*Gamma*-like *Epsilon*-like	MLV, FELV, WDSV	2341	27 C 25 NC	MMLV-like	HERV-T
					HERVERI	7.1.1.1 HERV-E, HERV3, HERV1, HERV-I
					HERVW9	HERV-W, HERV9
					HERVIPADP	HERVIP, HERVADP
					HERVHF	HERV-H, HERV-H48, HERV-FA, HERV-FB, HERV-FC, LTR46
					HERVFRD-like	HERV-FRD, PRIMA41, PABL, HERV4
					HEPSI	HEPSI2, HEPSI3, MER65, PRIMA4
					HUERSP	HUERSP1, HUERSP2, HUERSP3
Class II	*Beta*-like	MMTV, MPMV, JSRV	598	10 C 0 NC	HERV-K	HML1, HML2, HML3, HML4, HML5, HML6, HML7, HML8, HML9, HML10
Class III	*Spuma*-like	SFV	216	2 C 5 NC	HSERVIII	HERV-S, HERV-L
Uncertain	*Erranti*-like	Gipsy RV	2	0 C 1 NC	–	–
Unclass	–	–	16	–	–	–
Total			3,173	39 C 31 NC	–	–

*MLV, murine leukemia virus; FELV, feline leukemia virus; WDSV, walleye dermal sarcoma virus; MMTV, mouse mammary tumor virus; MPMV, Mason-Pfizer monkey virus; JSRV, jaagsiekte sheep retrovirus; SFV, simian foamy virus; C, canonical; NC, non-canonical*.

a *Exogenous retroviral species representative of the genus*.

## Env pleiotropic nature: from retroviral functions to physiological roles

(H)ERV Envs gained considerable attention due to the cooptation of some of them during eutherian mammal evolution, providing important biological activities to placenta development and pregnancy-related functions. Here we will only focus on the Env proteins relevant to human physiology, but it is worth noting that many Envs encoded by different (H)ERVs have been domesticated independently in different times and species, representing a fascinating example of convergent evolution (Lavialle et al., 2013).

**Figure 3:**
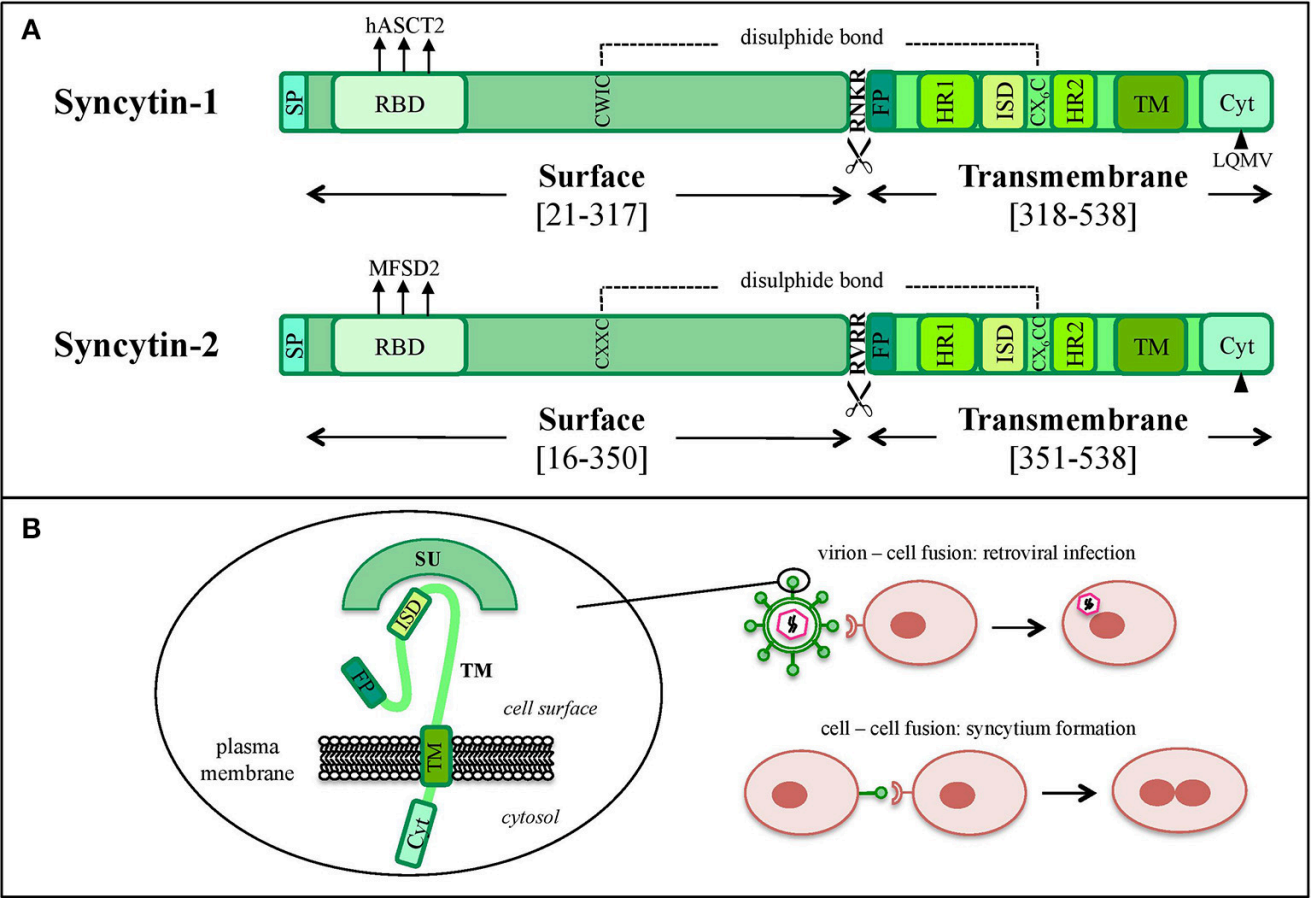
Graphical comparison between syncytin-1 and syncytin-2 proteins. **(A**) Schematic comparison of HERV-W syncytin-1 and HERV-FRD syncytin-2. The positions of Surface (SU) and Transmembrane (TM) subunits as well as the main functional and regulatory domains relevant for the protein's physiological activities are reported: signal peptide (SP), receptor binding domain (RBD) and the relative main cellular receptor, fusion peptide (FP), fusion core N- and C-terminal heptad repeats (HR1 and HR2, respectively), immunosuppressive domain (ISD), transmembrane motif (TM), intracytoplasmic tail (CYT) and the relative specific deletion for protease-independent activation (black triangle). The sites involved in SU and TM disulphide bond (dashed line) and furin cleavage (scissor symbol) are also indicated. Please see the text for more details about the individual domain functions. **(B)** Simplified model of the protein's structural configuration and fusogenic role when inserted into viral or cellular membranes.

In the retroviral life cycle, Env glycoproteins mediate the entry into the host cell. *env* encodes for a precursor that is cleaved into a SU subunit, constituting the viral antireceptor, and a TM subunit, holding fusogenic and immunosuppressive activities (Figures 1A, 3A,B). SU-TM heterodimers are assembled at the cellular membrane to form Env trimers, acquired by the viral particles during their budding. In the presence of a susceptible cell, the Env SU antireceptor binds the correspondent receptor on the cellular membrane, mediating the insertion of TM fusion peptide (FP) for membranes fusion and cytoplasmic release of the nucleocapsid. Env expression on the infected cell's surface also compete for receptor occupation, preventing superinfection of the same cell by multiple retroviruses, as shown for ERV-derived Env proteins in mouse (Best et al., 1997) and sheep (Varela et al., 2009). In addition, Env proteins present on the cell membrane can bind the correspondent receptors on uninfected cells, mediating membrane fusions and syncytia formation (Figure 3B). This mechanism is involved in the activity of coopted Env, namely “syncytins,” which similarly determine the fusion of the blastocyst's peripheral cells. Cytotrophoblasts form therefore a highly invasive layer, the syncytiotrophoblast, which invades the maternal uterine decidua constituting the outer placenta surface, being fundamental for embryo implantation and trophic exchanges.

In addition to fusogenicity, the retroviral Env TM subunit is known to possess an immune modulatory activity, possibly due to the presence of a putative immunosuppressive domain (ISD) (Figures 3A,B). In exogenous retroviruses this immune suppression activity is normally used to counteract the host antiviral responses (Mangeney and Heidmann, 1998; Blaise et al., 2001) and, in the case of HERVs, Env-mediated immune modulation has been coopted on occasion for the physiological maternal tolerance during pregnancy (Mangeney et al., 2007; Lavialle et al., 2013). In the latter, a well-evolved immune balance must allow fetal trophoblast invasion avoiding the rejection of paternal antigens (Ags), but should also maintain its activity in counteracting viral and bacterial infections. In particular, pregnancy is characterized by the suppression of cellular immunity that could stimulate cytotoxic processes and be harmful to the fetus. In this context, retroviral Envs have been suggested to inhibit maternal Th1 cytokine production (TNF-α, IFN-γ, and IL-2), leading to a shift toward the anti-inflammatory Th2 cytokines response (IL-4, IL-5 and, especially, IL-10) (Haraguchi et al., 1992, 1995; Tolosa et al., 2012).

Due to their relevant roles for human physiology, *syncytin* genes have been subjected to a positive selection along primate evolution, as suggested by the limited human polymorphisms and the high conservation of non-human primates' homologous loci (Esnault et al., 2013; Lavialle et al., 2013).

### HERV-W syncytin-1

The first Env characterized for its domestication is syncytin-1, encoded by a HERV-W provirus in locus 7q21.2 (ERVWE1) that was acquired by primates ~25 millions years ago (Mallet et al., 2004). ERVWE1 is coding-defective for *gag* and *pol*, albeit retaining an *env* ORF producing a protein with pregnancy-related functions (Blond et al., 2000; Mi et al., 2000).

**Figure 4:**
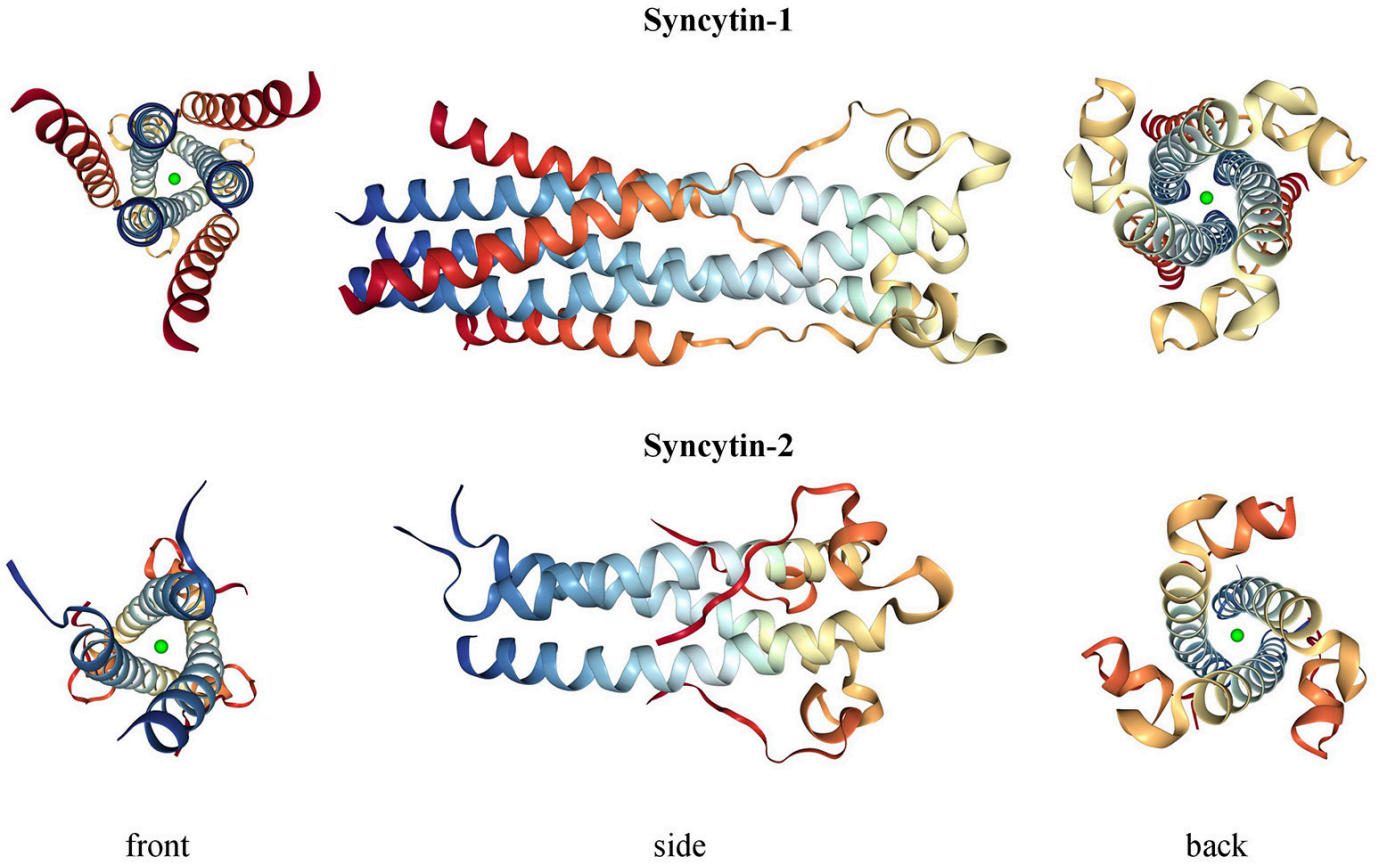
3D structure of syncytin-1 and syncytin-2 fusion subunits. Crystal structures were downloaded from RCSB Protein Data Bank (PDB) (Berman, 2000) at www.rcsb.org and are registered with the following identifiers: 5HA6 (syncytin-1, DOI: 10.2210/pdb5ha6/pdb) and 1Y4M (syncytin-2, DOI: 10.2210/pdb1y4m/pdb).

Syncytin-1 is a 73 kDa glycosylated protein composed of 538 amino acids (aa) and presenting an N-terminal signal peptide (SP, aa 1-20), a SU subunit (aa 21-317), a TM subunit (aa 318-538) and various functional domains for the protein maturation and activity (Gimenez and Mallet, 2008) (Figure 3A). In syncytin-1 precursor, SU and TM domains associate into a homotrimer through the TM N- and C-terminal heptad repeats (HR1 and HR2, aa 352-392 and 407-440, respectively) (Gimenez and Mallet, 2008). In this homotrimer, each precursor is cleaved by cellular furine proteases at the conserved SU/TM RKNR site, producing mature SU (gp50) and TM (gp24) subunits (Gimenez and Mallet, 2008). The latter remain linked through a disulphide bridge between SU CWIC and TM CX_6_CC motifs. Once located at the cellular membrane, SU N-terminal receptor binding domain interacts with the type D mammalian retrovirus receptor hASCT2 (human sodium-dependent neutral amino acid transporter type 2) (Lavillette et al., 2002; Cheynet et al., 2006). This activates syncytin-1 fusogenic activity, hold by the TM subunit and involving its hydrophobic FP (aa 320-340), the fusion core (composed of HR1 and HR2) and the TM C-terminal intracytoplasmic tail (CYT, aa 471-538) (Gimenez and Mallet, 2008). In particular, while in retroviral Envs the fusogenic activity requires the removal of an inhibitory peptide by viral proteases, syncytin-1 CYT presents a deletion of four aa (LQMV, after aa 485) that makes the protein constitutively competent for fusion (Bonnaud et al., 2004). The recent analysis of 16 HERV-W *env* ORFs and the correspondent putative proteins revealed their defectiveness as compared to syncytin-1, showing mutations in all sites relevant to fusogenic activity (Grandi et al., 2016). The syncytin-1 crystal structure was obtained for the sole fusion subunit (Figure 4), confirming the presence of a trimeric structure organized by hydrophobic α-helices association (Gong et al., 2005) (Figure 4). It has been suggested that, after the FP insertion, HR1 and HR2 associate in an antiparallel manner to form a stable α-helical trimer of heterodimers, i.e., a homotrimeric coiled coil complex (Gong et al., 2005).

Syncytin-1 was shown to induce syncytia through the interaction with hASCT2 receptor (Blond et al., 2000; Mi et al., 2000). Besides this, syncytin-1 is able to bind human divergent receptors (hASCT1) and even murine unglycosylated orthologs (mASCT1 and mASCT2), showing a low restriction that is likely due to a strong selective pressure throughout evolution (Lavillette et al., 2002). Syncytin-1 specifically localizes to the villous and extravillous trophoblasts, where its fusogenic activity has been coopted for the development of the placental syncytiotrophoblast (Blond et al., 2000; Mi et al., 2000; Malassiné et al., 2005). Syncytin-1 is also involved in the syncytiotrophoblast homeostasis, modulating cytotrophoblast differentiation, proliferation and survival by fostering the G1/S transition (Frendo et al., 2003; Huang et al., 2013b, 2014). In such regulation, cyclic AMP (cAMP), besides modulating the kinases involved in trophoblast fusion and differentiation (Keryer et al., 1998), controls a cAMP-responsive element (CRE) in ERVWE1 LTR (Frendo et al., 2003). Such cAMP stimulation promotes ERVWE1 basal expression, while an upstream tissue-restricted enhancer within an adjacent MaLR solitary LTR ensures high placenta-specific production (Prudhomme et al., 2004). This bipartite promoter is moreover under a joint regional epigenetic control, being hypomethylated with stage-dependent profiles in cytotrophoblasts (Gimenez et al., 2009) and being instead hypermethylated in non-placental tissues (Matousková et al., 2006). Aside from cAMP stimulation, the 5′-flanking region of ERVWE1 presents two binding sites for the chorion-specific transcription factor GCM1, required for placental development and shown to increase syncytin-1 expression and fusogenicity in trophoblasts, but not in other cells (Yu et al., 2002).

Syncytin-1 was also thought to have a role in maternal immune tolerance (Blond et al., 2000; Mi et al., 2000; Malassiné et al., 2005), as demonstrated for the ISD of a murine (Mangeney and Heidmann, 1998) and a primate (Blaise et al., 2001) retrovirus. Further studies in mice suggested instead the absence of such activity (Mangeney et al., 2007). However, syncytin-1 was able to inhibit Th1 cytokine production in human blood, suggesting a possible role in the shift from Th1 to Th2 cytokines occurring during pregnancy (Tolosa et al., 2012). Likewise, a physiological role of syncytin-1 was proposed in the upstream fertilization process: in fact, both syncytin-1 and hASCT2 receptor are expressed in human gametes, showing temporal and spatial appearance in line with a role in oocyte and sperm fusion (Bjerregaard et al., 2014).

Overall, syncytin-1 plays a pivotal role in placental morphogenesis and homeostasis through a well-evolved balance of fusogenic and non-fusogenic functions, having in addition some possible immunomodulatory activity during pregnancy.

### HERV-FRD syncytin-2

Similarly to syncytin-1, another Env encoded by a HERV-FRD provirus in locus 6p24.1 showed placenta-specific expression and syncytia induction in cultured cells, being therefore named syncytin-2 (Blaise et al., 2003). Syncytin-2 locus is found in both *Catarrhini* and *Platyrrhini* species, having been acquired >40 million years ago (Blaise et al., 2003).

Syncytin-2 is homologous to syncytin-1, being a ~73 kDa glycosylated protein of 538 aa expressed as a precursor that associates to form homotrimeric complexes (Renard et al., 2005; Chen et al., 2008; Cui et al., 2016) and presenting the same domains (Figure 3A): an N-terminal SP (aa 1-15), a SU subunit (aa 16-350) and a TM subunit (aa 351-528). SU and TM precursors are cleaved by a cellular furin protease at RVRR cleavage site (aa 347-350) and remain covalently associated through a disulphide bond between SU CX_2_C (aa 43-46) and TM CX_6_CC (aa 431-439) motifs. The TM subunit harbors the putative FP (aa 354-374), an ISD (aa 414-430), a TM domain (aa 479-499) and a C-terminal CYT domain (aa 500-538) missing the LQMV inhibitory motif in the cleavage site. Syncytin-2 crystal is also available for the fusion subunit only, being a homotrimer organized by associated HR1 and HR2 hydrophobic α-helices followed by an ISD loop and an extended domain antiparallel to the coiled-coil (Renard et al., 2005) (Figure 4).

Syncytin-2 expression is limited to the villous cytotrophoblasts (Malassiné et al., 2007), whereas its receptor, the transmembrane protein MFSD2 (major facilitator superfamily domain containing 2), is found specifically in the placental syncytiotrophoblast (Esnault et al., 2008). Syncytin-2 is thus responsible for a polarized fusion that drives the merging of individual cytotrophoblasts to the syncytiotrophoblast, sustaining its growth and regeneration in concert with syncytin-1 (Esnault et al., 2008). In addition, the syncytin-2 ISD is highly preserved and shows strong immunosuppressive activity, possibly providing immune tolerance to the fetal allograft (Mangeney et al., 2007). As for syncytin-1, syncytin-2 is epigenetically regulated at the proviral 5′LTR, being hypomethylated in placenta and hypermethylated in other cells (Gimenez et al., 2009; Liang et al., 2010); and it is further controlled by GCM1 that binds syncytin-2 and MFSD2A promoters stimulating placental cell-cell fusion (Liang et al., 2010).

### Other HERV-derived env proteins

Besides syncytins, other HERV Envs are expressed in normal conditions. This suggested their possible involvement in human physiology, even if no conclusive demonstrations have been reached yet.

The *env* gene of an ERV3 sequence in locus 7q11.21 contains a functional ORF (Boyd et al., 1993; Andersson et al., 2002, 2005) and ERV3 Envs have been detected in various human tissues, showing substantial expression in placenta (especially at the cyto/syncytiotrophoblastic layer), in the reproductive trait and in cells undergoing fusion either in physiological (myocardium, skeletal muscle) or pathological conditions (macrophages, tumors) (Fei et al., 2014).

HERV-K(HML2) Env expression has been detected in villous and extravillous cytotrophoblasts during the whole period of gestation, even if the protein has neither been found at any gestational time in placental syncytiotrophoblast nor linked to a specific locus of origin (Kämmerer et al., 2011). Furthermore, type II HML2 Rec proteins were recently found to be upregulated during embryogenesis, specifically stimulating an interferon (IFN)-induced viral restriction factor (IFITM126) in epiblast and embryonic stem cells (Yan et al., 2013; Grow et al., 2015). This led to the intriguing hypothesis that Rec and the associated HML2 transcripts might be sensed in the cytosol, stimulating an innate anti-viral response able to broadly inhibit embryonic viral infections (Grow et al., 2015). In the same embryonic cells, Rec was shown to interact with ~1,600 cellular mRNAs and to influence their ribosome occupancy, further suggesting a physiological role during early development (Grow et al., 2015).

Contrary to syncytins, the HERV Env encoded by a HERV-F provirus in locus 21q22.3 has been found to inhibit fusogenicity in mammals and therefore named suppressyn (Sugimoto et al., 2013). Suppressyn is a 160 aa polypeptide with placenta-specific expression, corresponding to the Env N-terminal portion and including a putative SP and a SU subunit with a premature stop codon upstream the SU/TM cleavage site (Sugimoto et al., 2013). The protein was shown to compete for the binding to syncytin-1 receptor and to significantly reduce its fusogenicity, possibly representing the first *env*-derived restriction factor found in *Catarrhini* primates (Sugimoto et al., 2013). Of note, the defective structure of this HERV-derived Env, lacking a portion of SU and the whole TM subunit, confirms that not only full-length but also truncated proteins can have some biological significance to the host.

### HERV env and oncogenesis: passive bystanders or active contributors?

**Figure 5:**
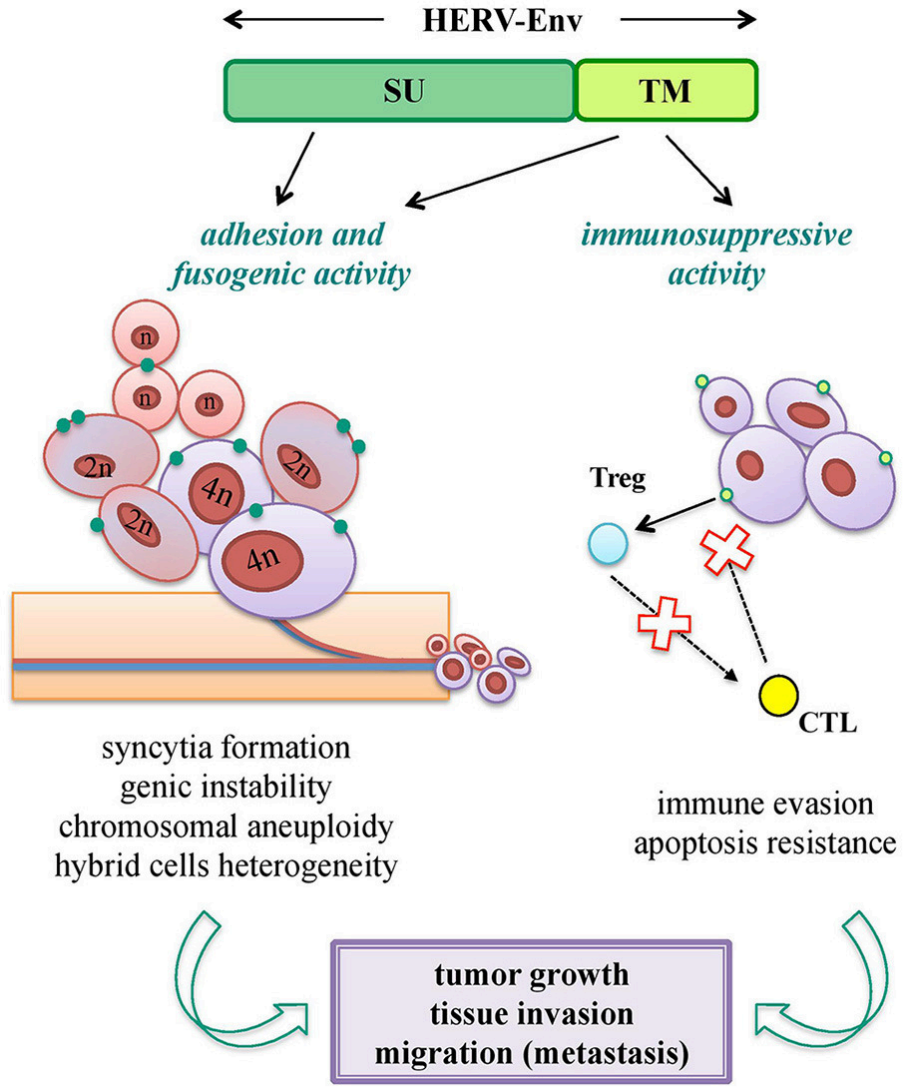
Pro-oncogenic potential of HERV-derived Env biological functions. Both SU and TM Env subunits can potentially trigger tumorigenic mechanisms through the same biological activities involved in their viral and physiological functions. Adhesion (SU) and fusogenic (TM) functions can lead to cell-cell fusion and syncytia formation, sustaining tumor genic and chromosomal instability; while immunosuppressive activity (TM) can mediate or facilitate the tumor cell immune-evasion, preventing cytotoxic (CTL) and apoptotic responses. All these processes can thus have an impact on tumor growth and in the subsequent process of tissue invasion and migration.

Oncogenesis is a multistep process hypothesized to be the result of a complex interplay between inherited and environmental factors, including viral infections. Nevertheless, while the oncogenic properties of exogenous retroviruses are well-known, HERV sequences have a more uncertain pathological significance, being expressed in many tissues without pathological consequences. Hence, a major obstacle is to properly evaluate whether their presence has a direct role in the disease onset, or if it is just an indirect product of transformation. In fact, tumors show a general epigenetic dysregulation, known to strongly and non-specifically liberate retrotransposon expression. Overall, different HERV-mediated mechanisms of oncogenesis have been proposed, including the ones not requiring any expressed product. Concerning HERV Env proteins, even if not confirmed yet as oncogenic agents, they have been suggested to support tumorigenesis through the same biological activities domesticated for physiological purposes: fusogenicity and immunosuppression (Figure 5).

Cell-cell fusion is known to occur physiologically in certain tissues (e.g., myocardium, skeletal muscle, placenta) and is frequently observed under pathogenic stimuli, such as inflammation and cancer. Tumor cells are in fact able to fuse with each other and with non-transformed cells, and such a process is involved in cancer progression, metastasis and chemoresistance (Walker et al., 2009; Berndt et al., 2013; Bastida-Ruiz et al., 2016) (Figure 5). Cellular fusion is also an important source of malignant cell heterogeneity and genetic instability, leading to polyploidy and aneuploidy (Bastida-Ruiz et al., 2016) (Figure 5). Thus, given that cancer cells show migration and invasion features very similar to the ones of syncytiotrophoblast, domesticated HERV Env fusogenicity could stimulate the uncontrolled cell fusion in tumors, underlining the need for a strict regulation.

In addition, the ISD of HERV Envs may support tumor progression, abrogating the anti-oncogenic cytolytic immune response (Kassiotis and Stoye, 2017) (Figure 5). This hypothesis is sustained by the strong evidence of tumor promotion by the Env ISD of animal ERVs (Ruprecht et al., 2008b).

Apart from fusogenic and immunosuppressive activities, which can be held by any functional HERV Env, the HERV-K(HML2) sequences could sustain transformation through their accessory proteins Np9 and Rec, proposed to be HERV-derived oncogenes.

### HERV-W

A large body of studies reported the HERV-W group RNA and protein upregulation in human cancers, often with no information about the originating loci and the eventual influence of tumor epigenetic dysregulation on their expression (Stauffer et al., 2004; Yi et al., 2004; Bjerregaard et al., 2006; Kim et al., 2008; Díaz-Carballo et al., 2015, and reviewed in Grandi and Tramontano, 2017). Of note, in the case of HERV-W sequences, the hyperexpression prompted by hypomethylated environments could possibly trigger the *de novo* retrotransposition of mRNA sequences (Grandi and Tramontano, 2017). This event has been ancestrally responsible for the formation of HERV-W processed pseudogenes, accounting for ~2/3 of the actual group members (Grandi et al., 2016) and occurred through the L1-mediated reverse transcription and mobilization of HERV-W RNA transcripts (Costas, 2002; Pavlícek et al., 2002). Thus, given that (i) our genome still contains 80–100 retrotransposition-competent L1, and that (ii) L1 reactivation has been broadly reported in human malignancies (Hancks and Kazazian, 2016; Scott and Devine, 2017) and implicated in metastasis and cancer progression (Papasotiriou et al., 2017), the *de novo* L1-mediated insertion of HERV-W processed pseudogenes could possibly further contribute to tumor genetic instability (Grandi and Tramontano, 2017).

In breast and endometrial carcinomas, syncytin-1 fusogenicity has been proposed to mediate cancer cell fusion with other tumoral or normal cells (Bjerregaard et al., 2006; Strick et al., 2007), potentially altering their biological behavior and sustaining tumor progression. Particularly, human breast cancer (hBC) cell lines that express syncytin-1 on the cellular membrane are able to fuse with endothelial cells presenting hASCT2 receptor (Bjerregaard et al., 2006). Such Env expression on hBC cells was found in ~40% of patients, being related to the rate of recurrence and survival and is thus proposed to be a prognostic marker (Larsson et al., 2007). The common occurrence of giant syncytial cells was similarly observed in endometrial carcinomas, whose development is known to be linked to hormone replacement therapies. Thus, both the fusogenic and the steroid-driven nature of this tumor were investigated in relation to syncytin-1 expression. Even if syncytin-1 was upregulated also in benign endometrial specimens, the highest expression was observed in carcinoma tissues, being induced by steroid hormones due to the presence of an hypomethylated estrogen responsive element (ERE) in ERVWE1 5′LTR (Strick et al., 2007; Strissel et al., 2012).

In some other instances, HERV-W Envs have been linked to tumor development due to their interference with crucial cellular pathways. For example, syncytin-1 was upregulated in >75% of bladder urothelial carcinomas, increasing proliferation and viability of immortalized uroepithelial cells, and a recurrent single nucleotide substitution in ERVWE1 3′LTR was shown to drive the binding of c-myb transcription factor, possibly empowering syncytin-1 promoter activity in this malignancy (Yu et al., 2014). In neuroblastoma, such dysregulation was shown to involve the cAMP-pathway, already mentioned for its regulatory role on the syncytin-1 promoter (Frendo et al., 2003) and cytotrophoblast differentiation (Keryer et al., 1998). Particularly, in neuroblastoma cell lines, the HERV-W Env transfection mediated an augmented phosphorylation of the activating transcription factor CREB (CRE-binding protein), leading to the hyperactivation of an ion channel (SK3, small conductance Ca2+- activated K+ channel protein 3) already known to be involved in neuron excitotoxicity and neurological diseases (Li et al., 2013).

Furthermore, a few studies reported the presence of syncytin-1 in other types of malignancies, without however characterizing its possible contribution to cancer. Syncytin-1 was detected in colorectal carcinomas, being confined to the tumor areas and hyperexpressed in villar and intervillar regions as well as in large intestine crypts (Larsen et al., 2009; Díaz-Carballo et al., 2015). In mycosis fungoides (the most common primary cutaneous T-cell lymphoma), 50% of patients expressed syncytin-1 in infiltrating lymphocytes, while no expression was detected in patients presenting benign T cell infiltrates (Maliniemi et al., 2013). In a third study on leukemia, >2/3 of samples showed abnormal syncytin-1 expression, and the protein was detected in the blood cells of patients but not healthy controls (Sun et al., 2010).

Finally, in a few cases, syncytin-1 was contrarily downregulated in cancer, possibly suggesting its positive prognostic value in certain tumors. Accordingly, pancreatic adenocarcinoma samples showed reduced syncytin-1 expression concomitant to ERVWE1 LTRs hypermethylation (Lu et al., 2015), and B16F10 melanoma cells stably expressing syncytin-1 were significantly limited in proliferation and invasion (Mo et al., 2013).

### HERV-FRD

While many studies were devoted to syncytin-1 expression in cancer, very few studies investigated the second domesticated Env protein, HERV-FRD syncytin-2 (Table 2). In the first study in hBC cells, the ectopic expression of GCM1 led to the hypomethylation of syncytin-2 5′LTR, stimulating the protein expression and fusogenicity (Liang et al., 2010). In a second study, syncytin-2 overexpression was shown in endometrial tumoral and pre-tumoral lesions, being significantly associated with the disease stage and histological grading (Strissel et al., 2012). Finally, a significant upregulation of syncytin-2, along with syncytin-1, has been reported in colon adenocarcinoma samples (Díaz-Carballo et al., 2015).

**Table 2:** Main molecular evidence of putative pro-oncogenic activity of HERV-derived Env.

**Group**	**Env protein**	**Tumor type**	**Molecular mechanism**	**Oncogenic effect**	**References**
HERV-W	Syncytin-1 (7q21.2)	Breast	Fusogenicity	Cell-cell fusion	Bjerregaard et al., 2006
		Endometrial	Fusogenicity	Cell-cell fusion	Strick et al., 2007; Strissel et al., 2012
		Neuroblastoma	Cellular pathway alteration	SK3 hyperactivation, excitotoxicity	Li et al., 2013
		Bladder urothelial cells	Unknown	Increased cell proliferation and viability	Yu et al., 2014
HERV-FRD	Syncytin-2 (6p24.1)	Breast	Ectopic GMC1-induced fusogenicity	Cell-cell fusion	Liang et al., 2010
		Endometrial	Fusogenicity	Cell-cell fusion	Strissel et al., 2012
HERV-K (HML2)	Env	Breast	Stimulation of cellular pathways playing key roles in cancer (*EGFR, TGFB1, NF-κB, c-myc, p53, Ras, p-RSK, p-ERK1/2*…)	Increased cell proliferation, transformation, migration and invasion	Zhou et al., 2016; Lemaître et al., 2017
	Rec	Germ cell	Interaction with PLZF abrogates *c-myc* repression	Increased cell proliferation, apoptosis abrogation	Denne et al., 2007
			Interaction with TZFP and hSGT abrogates the repression of AR and AR-regulated genes (e.g., *c-myc*)	Increased cell proliferation, apoptosis abrogation, stimulation of Rec expression	Kaufmann et al., 2010; Hanke et al., 2013a
	Np9	Germ cell	Interaction with PLZF abrogates *c-myc* repression	Increased cell proliferation, apoptosis abrogation	Denne et al., 2007
		–	Interaction with LNX ubiquitin ligase	Perturbation of Numb/Notch pathway	Armbruester et al., 2004
		–	Interaction with MDM2 ubiquitin ligase	Reduced MDM2-mediated proteasomal degradation of p53	Heyne et al., 2015
		Lymphoma (EBV-transformed B cells)	Interaction with EBV EBNA2	Downregulation of viral promoter activation and transcription factors binding	Gross et al., 2011
		Leukemia	Upregulation of pERK, c-Myc, β-catenin and Notch1	Increased cell proliferation, faster and increased tumor growth	Chen et al., 2013

*HERV-derived Env proteins for which a specific pro-oncogenic activity and/or a precise effect on cellular transformation have been characterized are included*.

### HERV-K

The HERV-K supergroup, composed of the Human MMTV-like (HML) groups 1–10 (Table 1), probably constitutes the most investigated HERV ensemble in relation to carcinogenesis. Particularly, in-depth attention was devoted to HERV-K(HML2) group, often generically referred to just as HERV-K. This group includes the evolutionarily youngest HERV sequences, showing a remarkably recent activity that led to the formation of both human-specific loci (i.e., present in humans but not in non-human primates) (Subramanian et al., 2011) and unfixed insertions within human population (Marchi et al., 2014). Moreover, as mentioned above, HML2 Np9 and Rec accessory proteins have been suggested to be oncogenes.

#### HERV-K(HML2)

**Figure 6:**
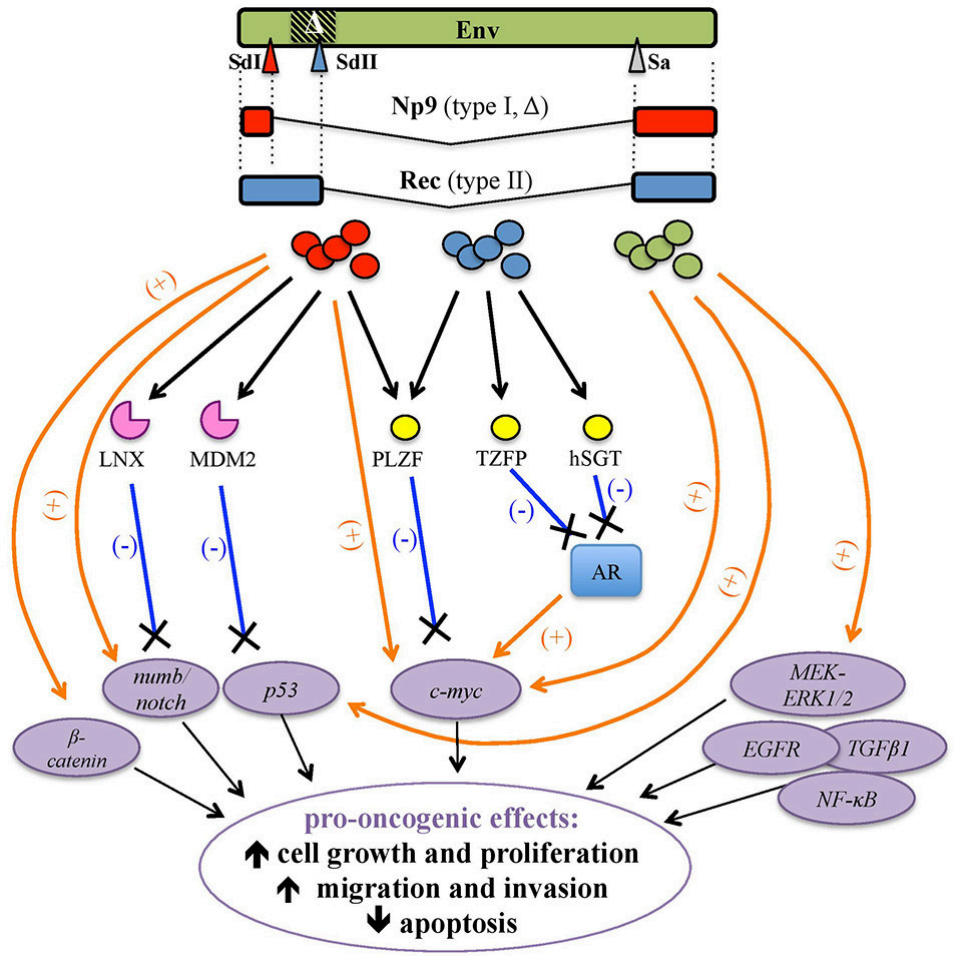
Pro-oncogenic effects associated with HERV-K(HML2) Env proteins and Rec and Np9 accessory variants. HERV-K(HML2) Env proteins (green) as well as both type I and type II *env*-derived accessory splicing variants, namely Np9 (red, splicing donor site I) and Rec (blue, splicing donor site II), respectively, have been reported to potentially trigger transformation by the positive stimulation (orange arrows) of cellular transcription factors and pro-oncogenes (violet circles) and/or through the interaction with proteins controlling their degradation (pink) or repression (yellow), leading to the lack of negative regulation (blue lines). The main potential effects on tumor growth and progression are indicated.

HML2 *env* expression attracted significant attention in the tentative link to tumorigenesis, either as Env or as the above accessory variants Np9 and Rec (Figures 1B, 6).

##### Env

The upregulation of HML2 *env* in hBC cells and tissues (Wang-Johanning et al., 2001, 2003; Zhao et al., 2011) together with the link between MMTV and mouse mammary carcinoma (Bittner, 1936; Matsuzawa et al., 1995) greatly prompted the search for a possible involvement of HML2 Env in hBC. The protein is known to be present at different levels in both *in situ* and invasive hBC tissues, stimulating cellular and humoral immunity (Wang-Johanning et al., 2008; Zhao et al., 2011), and its overexpression has been linked to hBC stage, grade, p53 mutation status and metastatic spread, having thus a possible prognostic value (Zhao et al., 2011; Zhou et al., 2016). Of note, monoclonal antibodies (Abs) against HML2 Env, which were shown to inhibit the proliferation of hBC cells and the growth of xenograft tumors in mice, are being investigated as anti-cancer agents (Wang-Johanning et al., 2012). Overall, while many findings reported the presence of either HML2 Env in diseased tissues or a specific immune responses against it in hBC patients, the molecular mechanisms involving the protein in hBC oncogenesis are still not fully clarified. A recent study showed that HML2 Env knockdown was able to inhibit hBC cell proliferation, migration and invasion; affecting various cellular networks playing key roles in cancerogenesis (*EGFR, TGFB1, NF-*κ*B, c-myc*, and *p53*) and impairing tumor-associated gene expression (*ras, p-RSK*, and *p-ERK*) (Zhou et al., 2016) (Figure 6). Accordingly, HML2 Env downregulation significantly reduced tumor formation and metastasis in mouse xenografts while, when overexpressed in hBC cells, the previously observed impairments in cellular networking, migration, invasion, and transformation were reverted (Zhou et al., 2016). Likewise, HML2 Env expression in non-transformed breast cells prompted epithelial to mesenchymal transition, leading to an increase in cell motility, migration and invasion (Lemaître et al., 2017). Mechanistically, such behavioral changes were mediated by HML2 Env through the activation of ERK1/2 MAPK pathway (Figure 6) and the stimulation of transcription factors associated with cancer aggressiveness (Lemaître et al., 2017).

Additional evidence of HML2 Env oncogenicity comes from intensive studies in melanoma. Both HML2-derived Env and Rec proteins have been detected at variable percentages in melanoma biopsies and cell lines, being instead generally absent in normal melanocytes (Muster et al., 2003; Büscher et al., 2005, 2006). Moreover, HML2 virus-like particles with RT activity but lacking infectivity were observed in melanoma cell cultures (Muster et al., 2003; Büscher et al., 2005, 2006; Serafino et al., 2009). HML2 Env was shown to trigger humoral immunity in ~20% of melanoma patients, and patients with anti-HML2 Abs had a decreased overall survival (Büscher et al., 2005; Hahn et al., 2008). Finally, HML2 Env inhibition decreased the fusion of cultured melanoma cells, suggesting a role in the formation of multinuclear cancer cells and in the onset of genetic heterogeneity conferring trophic and survival advantages to tumor cells (Huang et al., 2013a). In this regard, a pivotal role in melanoma development has been suggested for cancer subpopulations bearing stem cell properties (Frank et al., 2010). CD133+ melanoma cell lines undergoing medium-induced phenotype-switching showed stemness features (enhanced proliferation, migration and invasion) concomitant with the activation of HML2 expression, thus suggested to have a role in cellular plasticity (Argaw-Denboba et al., 2017). Similarly, stress conditions stimulating the transition from an adherent to a non-adherent (and more malignant) phenotype led to a concomitant upregulation of HML2 expression, whose inhibition was in turn able to prevent the phenotype-switch (Serafino et al., 2009). Given that the observed morpho-behavioral changes are analogous to the ones arising from *BRAF* gene suppression in melanoma, commonly leading to the constitutive activation of MEK–ERK signaling, HML2 Env expression has been related to ERK activation, being downregulated after the inhibition of MEK or CDK4 (Li et al., 2010). In general, this augmented HML2 expression in melanoma could represent a tumor epiphenomenon, especially in the presence of impairments in cellular signaling and stress conditions altering the tumor transcriptional environment. In the case of melanoma, for example, UV irradiation is a known risk factor and has been shown to trigger HERV-K protein expression (Reiche et al., 2010; Schanab et al., 2011). Similarly, a melanoma-specific transcription factor is able to activate HML2 LTRs (Katoh et al., 2011) and the RB protein, a downstream mediator of BRAF–MEK–ERK signaling often altered in cancers, is a key regulator of DNA methylation that can influence HERV expression (Li et al., 2010).

HML2 Env was detected on the surface of ovarian cancer (OC) lines and patients' cells, showing a general correlation with the tumor histotype (Wang-Johanning et al., 2007; Rycaj et al., 2015). OC patients showed significantly higher titers of Abs against HML2 Env and specific T-cell cytotoxicity against autologous OC cells (Wang-Johanning et al., 2007; Rycaj et al., 2015). However, the similar Ab positivity found against HERV-E and ERV3 Envs (Wang-Johanning et al., 2007) together with the general hypomethylation of HERV sequences in OC (Iramaneerat et al., 2011) suggest that the increased HERV expression and Ab production could constitute (at least in part) a tumor epiphenomenon. However, even HERV proteins arisen from tumor-dependent upregulation can then participate in a multifactorial stimulation, contributing to cancer progression.

##### Rec

HML2 Rec is a functional homolog of HIV-1 Rev and HTLV Rex accessory proteins, protecting the retroviral transcripts from cellular splicing and enhancing their nuclear export (Magin et al., 1999). Thus, Rec shares with Rev and Rex the main structural and biological properties. First of all, to export viral RNAs, these proteins need to be imported from the cytoplasm to the nucleus through a specific nuclear localization signal (NLS) rich in basic amino acids (often arginines) that interacts with cellular import factors. Once inside the nucleus, the efficient binding to viral transcripts relies on the interaction between Rev/Rex/Rec and a specific responsive element in viral transcripts. These responsive elements, named RRE/RxRE/RcRE, respectively, can be located either within *env* (RRE) or in the 3′UTR (RxRE, RcRE) and show an highly structured and folded RNA organization (Magin et al., 1999; Magin-Lachmann et al., 2001). In the case of HML2 transcripts, RcRE presents four stem-loops essential for Rec- but not Rev- and Rex-mediated export, that does occur *in vitro* through discrete binding sites (Magin-Lachmann et al., 2001). The so-formed ribonucleoprotein multimers cooperate then with various host factors to stabilize the transcripts and mediate their nuclear export, competing in this way with the cellular splicing machinery. To do this, Rec/Rev/Rex presents a nuclear export signal (NES) rich in leucines that is recognized by cellular exportins, the most important of which is CRM1, to mediate the active egress of the protein with the associated unspliced RNAs to the cytoplasm (Fornerod et al., 1997; Fukuda et al., 1997; Neville et al., 1997; Ossareh-Nazari, 1997; Magin et al., 1999; Boese et al., 2000b). Besides CRM1, Rec can interact with Staufen-1, an ubiquitous protein involved in the cytoskeleton-mediated transport of ribonucleoprotein complexes, increasing the export and translation of Rec-associated mRNAs (Hanke et al., 2013b). Staufen-1 is also involved in RNA decay during cellular stress, recruiting non-essential mRNAs and accumulating them into stress granules or processing bodies, to prevent their translation or mediate their degradation, respectively. Accordingly, in stressed cells, Rec co-localizes with Staufen-1 in stress granules, leading to the block of viral RNA translation (Hanke et al., 2013b).

Besides these virus-specific regulatory functions, a pathological role of Rec was firstly suggested in human germ cell tumors (hGCT), due to the expression of HML2 *env* spliced mRNA variants (Löwer et al., 1993) and the development of anti-HML2 Env Abs in ~85% of patients (Sauter et al., 1996). Such putative oncogenic properties were then attributed to the Rec splicing variant, given that tumor development was observed in nude mice receiving injections of HML2 Rec but not in the ones injected with the full-length Env (Boese et al., 2000a). HML2 Rec expression in transgenic mice was moreover able to induce *in situ* testicular carcinomas, the predecessor lesion of GCT (Galli et al., 2005). At the molecular level, Rec was shown to interact with human tumor suppressor PLZF (promyelocytic leukemia zinc-finger protein) (Boese et al., 2000a), known to be involved in leukemia development. Rec binding to PLZF abolished the PLZF-mediated transcriptional repression of *c-myc*, stimulating cell growth and proliferation (Denne et al., 2007) (Figure 6). Besides PLZF, Rec can form complexes with the androgen receptor (AR) (Kaufmann et al., 2010) as well as with its negative regulators: the PLZF-related testicular zinc-finger protein co-repressor (TZFP) (Kaufmann et al., 2010) and the human small glutamine-rich tetratricopeptide repeat-containing protein co-chaperone (hSGT) (Hanke et al., 2013a). In particular, Rec can associate in a trimeric complex with TZFP and AR, overcoming the former stimulation by the latter (Kaufmann et al., 2010), and can also directly interact with hSGT, similarly abrogating its capacity to bind and suppress AR (Hanke et al., 2013a) (Figure 6). Such a pathway could be further sustained by hormone stimulation, including androgens: in this vicious cycle, the Rec-mediated suppression of AR negative regulation on the one hand enhances the expression of AR-dependent genes, leading to cellular proliferation and reduced apoptosis, and, on the other hand, it activates HML2 LTRs, further stimulating Rec production (Hanke et al., 2013a).

In hBC, anti-Rec Abs were detected in early-stage patients and suggested to be predictive of the disease progression (Wang-Johanning et al., 2013). It was proposed that Rec interaction with AR/TZFP/hSGT and the activation of *c-myc* can cooperate with AR-mediated dysregulation of HER2/HER3 signaling (Hanke et al., 2016). However, to the best of our knowledge, no specific molecular mechanisms of tumorigenesis in hBC have been demonstrated yet, and the pro-oncogenic activation of MAPK-ERK1/2 pathway observed for HML2 Env was instead absent when considering Rec (Lemaître et al., 2017).

##### Np9

HML-2 Np9 originates as an *env* shorter splicing variant (~9 kDa) associated with type I HML2 proviruses (Figure 1B) and, differently from the Rec protein that is normally found in the cytoplasm, it shows a predominant nuclear localization (Armbruester et al., 2002). Np9 expression was originally reported in transformed cells only (Armbruester et al., 2002) and has therefore been investigated for its oncogenic potential, even if subsequent studies revealed its physiological transcription in various healthy tissues (Schmitt et al., 2015).

First of all, the homology of Np9 and Rec in the 14 aa at the N-terminus drove the search for common cellular partners relevant to tumor transformation (Armbruester et al., 2002). Thus, Np9 was shown to bind PLZF in the nucleus, not through the N-terminal portion shared with Rec but with the first NLS (Denne et al., 2007), already shown to be critical for the protein nuclear localization (Armbruester et al., 2004). In this way, Np9 was suggested to potentially act as an oncoprotein, interfering with PLZF repression of *c-myc* (Denne et al., 2007) (Table 2, Figure 6).

Np9 was also shown to interact with two E3 ubiquitin ligases involved in the proteasome-dependent degradation of cellular proteins relevant to proliferation and transformation. Firstly, Np9 can bind LNX (Ligand of Numb protein X), which mediates Numb ubiquitylation and degradation (Armbruester et al., 2004). Numb is a crucial cell fate determinant and functions as Notch antagonist in the differentiation/proliferative pathway. Notch pathway is, in fact, an evolutionarily conserved signaling network regulating proliferation, differentiation and self-renewal of stem and progenitor cells, and its dysregulation has been involved in some tumors (Flores et al., 2014). While LNX-Numb interaction occurs in endosomes, Np9 competes for LNX binding directing the protein within nucleoli, affecting in this way LNX intracellular localization and Numb degradation, thereby promoting Notch signaling (Armbruester et al., 2004) (Table 2, Figure 6). Interestingly, Np9 interacts with the Epstein-Barr Virus (EBV) nuclear Ag EBNA2, which is considered to be a viral homolog of Notch due to its capacity to promote B-cell proliferation and immortalization (Gross et al., 2011). Particularly, Np9 nuclear binding to EBNA2 exerted a negative impact on the latter functions of promoter activation and binding to cellular transcription factors (Gross et al., 2011) (Table 2). In addition, Np9 was shown to bind another E3 ligase, MDM2, which has a pivotal role in the negative regulation of p53 transcription factor through its ubiquitylation for degradation or nuclear exclusion (Heyne et al., 2015). Np9 was shown to compete with p53 for the binding to MDM2, thus affecting the former proteasomal degradation and possibly supporting its uncontrolled activity as a further pro-oncogenic mechanism (Heyne et al., 2015) (Table 2, Figure 6).

Additional evidence of Np9 oncogenic activity was obtained in myeloid and lymphoblastic leukemia cells that were promoted in survival and growth by Np9 overexpression and, when injected in immunodeficient mice, led to faster tumor growth and increased tumor weight (Chen et al., 2013). Moreover, in leukemia cells expressing native Np9, the protein was shown to co-activate multiple leukemia-associated signaling pathways, inducing an aberrant upregulation of pERK, c-Myc and β-catenin and cleaving Notch1 with a subsequent decrease of Numb (Chen et al., 2013) (Table 2, Figure 6). Overall, the ability of Np9 to act as a molecular switch of multiple signaling pathways critical to cellular growth and proliferation could support a pro-oncogenic role of this protein.

#### HERV-K(HML6)

The sole finding linking HML6 Envs to human cancers was reported in a melanoma patient which presented an HML6 Ag expressed on tumor cells and targeted by cytolytic T lymphocytes (Schiavetti et al., 2002). Such Ag was encoded by a HERV-K(HML6) *env* gene located in chromosome 16, being expressed in ~85% of transformed melanocytes and generally absent in normal tissues, and was therefore named HERV-K-MEL (Schiavetti et al., 2002). HERV-K-MEL was proposed as a biomarker for melanoma onset, even if its expression was detected in the majority of benign nevi and in normal skin samples as well (Schiavetti et al., 2002). The authors suggested subsequently that HERV-K- MEL derived Ags, being targeted by patients' cytolytic T lymphocytes, could constitute a target for vaccination and anti-cancer approaches, as discussed below.

### Other HERV groups

Besides the above-discussed groups, other HERV Env proteins have been investigated by a few studies. Donor T lymphocytes infusion has curative effects for hematological malignancies, and has been shown to induce tumor regression in metastatic renal cell carcinoma patients. In tumor lines established from the latter biopsies, the onset of alloreactive T cells was observed and the recognized sequences had 100% homology with proteins encoded by a HERV-E locus on chromosome 6 (Takahashi et al., 2008). Such protein expression was up-regulated in the sole clear cell carcinoma variant, being promoted by the hypoxia-inducible transcription factor 2α as a consequence of von Hippel-Lindau factor (VHL) inactivation (Takahashi et al., 2008). Later on, the same HERV-E locus was shown to express a full-length *env*, similarly found in clear cell renal carcinomas only and detected in all patients harboring VHL deficiency (Haruta et al., 2015). The correspondent putative Env stimulated cytolytic T lymphocytes specifically recognizing the HERV-E-expressing carcinoma cells, with a possible immunotherapeutic application (Haruta et al., 2015). Similarly, a HERV-H *env* in chromosome X was found to be highly expressed in a subset of gastrointestinal cancers (Wentzensen et al., 2007) and the putative Env peptides showed the ability to stimulate autologous cytolytic T lymphocytes, leading to INFγ production and lysis of colorectal carcinoma cells (Mullins and Linnebacher, 2012a).

## HERV env immunopathogenic properties: possible roles in autoimmunity

**Figure 7:**
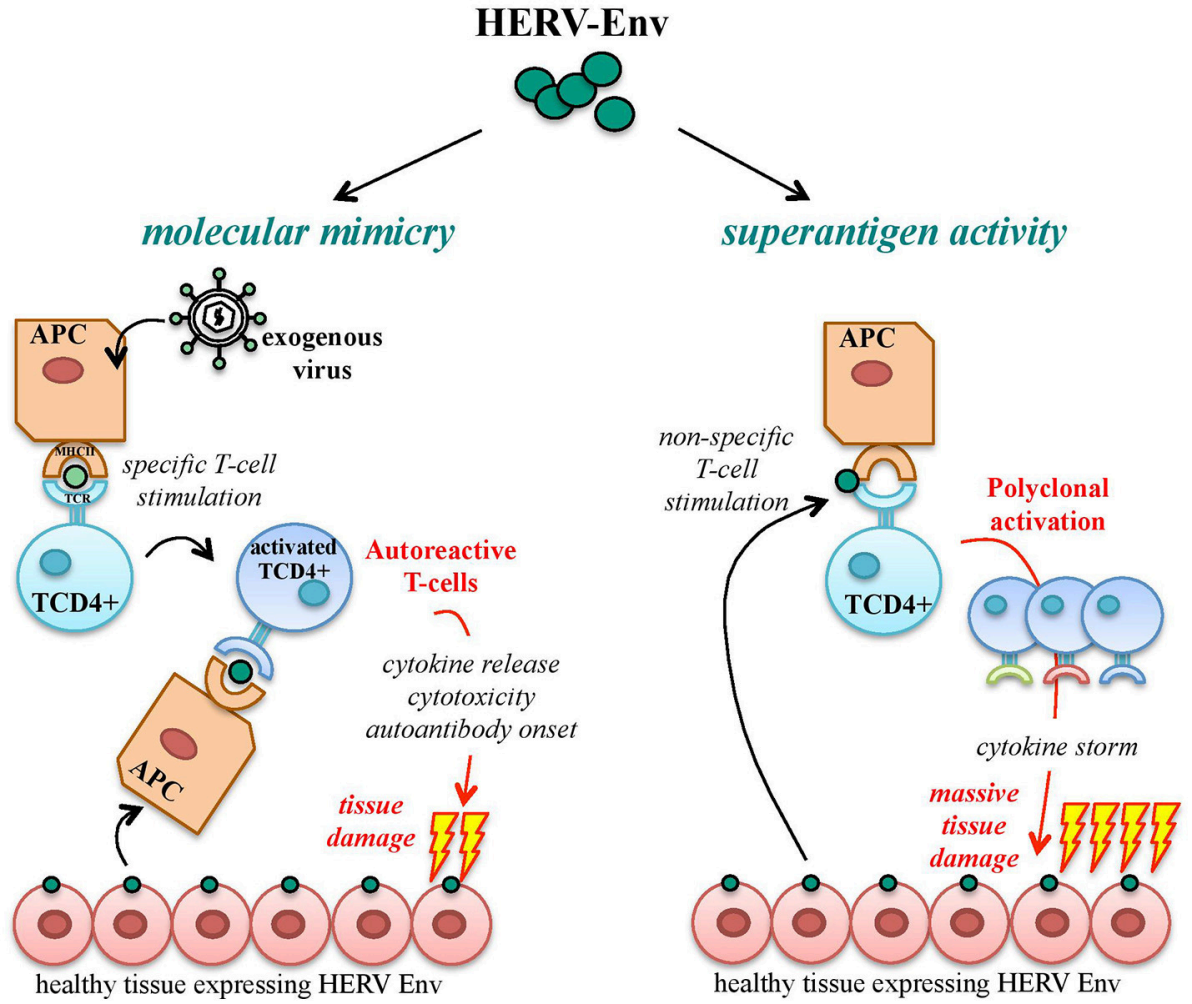
Pathogenic potential of HERV-derived Env proteins in autoimmunity. HERV-derived Env proteins have been implicated in the pathological stimulation of the host immunity mainly through two mechanisms: molecular mimicry and superAg activity. In the case of molecular mimicry, Ag-presenting cells (APCs) expose Env epitopes of an exogenous infectious agent by their major histocompatibility complex (MHC), stimulating the specific activation of T CD4+ lymphocytes. In the presence of similarity with HERV-derived proteins, endogenous Env Ags expressed on healthy cells can be cross-recognized by activated T cells, which trigger autoimmunity mechanisms by the release of proinflammatory cytokines and the stimulation of humoral and cellular adaptive responses, leading to tissue injury and destruction. In addition, HERV Env proteins can act as strong activators of the immune system with superAg function, prompting the non-specific stimulation of T lymphocytes. The consequent polyclonal expansion of reactive T cells can led to massive cytokine release, with extensive tissue damage and systemic life-threatening manifestations (shock, multi-organ failure).

A functional immune system is able to discriminate between foreign immunogenic Ags, stimulating effective immune responses, and self-Ags, for which immune tolerance becomes established during development. Autoimmunity defines a heterogeneous ensemble of multifactorial disorders sharing the loss of such tolerance. Its clinical manifestations include the activation of T helper lymphocytes and the onset of Abs and/or cytotoxic T cells directed against body components, leading to chronic inflammation and tissue damage. In theory, HERV products should be recognized as self-Ags, being stable components of the human genome highly expressed during development, when immune tolerance is acquired. Nevertheless, HERV expression is still able to trigger both innate and adaptive immunity, being subsequently investigated in a large number of autoimmune disorders. To explain such paradox, the most accepted model is that HERV Ags stimulate immunity due to their similarity to exogenous viral proteins, i.e., based on molecular mimicry (Trela et al., 2016) (Figure 7). In this way, HERV Ags normally expressed in healthy cells may be considered as pathogen associated molecular patterns (PAMPs) by the innate immunity pattern recognition receptors (PRRs), triggering inflammation and T helper lymphocytes differentiation and evoking cellular-mediated cytotoxicity and auto-Ab production (Hurst and Magiorkinis, 2015). Besides molecular mimicry, even in the absence of a specific immune recognition, HERV proteins can elicit the non-specific polyclonal activation of auto-reactive T lymphocytes, acting like super-Ags and inducing massive cytokine release with potentially life-threatening manifestations (shock, multi-organ failure) (Brodziak et al., 2011; Emmer et al., 2014) (Figure 7). Finally, as described for cancer, the presence of an altered epigenetic environment has been widely reported in autoimmunity too, and must be considered when investigating the upregulation of HERV expression and its possible pathological significance. Nevertheless, even the hypomethylation-dependent onset of HERV protein could provide immunopathogenic agents possibly suitable as biomarkers and therapeutic targets.

In autoimmune diseases, Env proteins are the most intensively investigated HERV products due to their remarkable immunopathogenic properties. Overall, the soundest evidence has been obtained about the HERV-W Env immunopathogenic potential in multiple sclerosis (MS), while the findings about other HERV Env contribution to autoimmune disorders are still quite controversial. However, as already mentioned for cancers, no HERV sequence or protein has been definitively associated with any autoimmune disease yet.

### HERV-W

The potential role of HERV-W sequences and their expression products in autoimmunity has been recently reviewed (Hon et al., 2013; Grandi et al., 2017). Concerning HERV-W Env proteins, the majority of studies investigated their role in MS and, only recently, in chronic inflammatory demyelinating polyradiculoneuropathy (CIDP) and type 1 diabetes.

#### Multiple sclerosis and other demyelinating diseases

MS is an autoimmune disease having as main signature the progressive demyelination of the central nervous system, with immunopathogenic manifestations sustained by alterations in both innate and adaptive immunity (Antony et al., 2011). As many autoimmune disorders, MS shows a complex and poorly understood etiology, which somehow prevented the development of specific therapeutic approaches. A great number of etiological determinants has been proposed, including genetic predisposition, environmental factors and various infectious agents (Perron and Lang, 2010; Libbey et al., 2013; Morandi et al., 2015) that likely participate to a multifactorial immunopathogenesis. The specific causes of demyelination and axon damage are still not fully clarified, even if inflammatory reactions prompted by cytokines, chemokines, prostaglandins, reactive oxygen species and matrix metalloproteinases are known to play a significant role (Antony et al., 2011). The initial link between the HERV-W group and MS was prompted by its sequence identity with MSRV (MS retrovirus), a putative exogenous element detected in a variable proportion of MS patients (Garson et al., 1998; Komurian-Pradel et al., 1999; Voisset et al., 1999). The origin and nature of MSRV is, however, still subject of debate (Blomberg et al., 2000; Ruprecht et al., 2008a; Voisset et al., 2008), and the MSRV sequences found in MS patients might have originated from the expression of individual HERV-W loci or by the recombination of different HERV-W transcripts (Schmitt et al., 2013; Grandi et al., 2016), known to often confound in the *in vitro* analysis of multicopy HERV sequences (Flockerzi et al., 2007). In the last three decades, many studies reported the presence of HERV-W transcripts in MS samples; HERV-W Ags in MS lesions or specific B and T cell immune responses against them in MS patients. Concerning HERV-W/MSRV Envs, even if the (variable) presence of such proteins in MS patients is undoubted, their precise role in the disease etiology is still to be defined. Nevertheless, their clear immunopathogenic potential—as shown through the use of MS animal models—led to their current exploitation as innovative therapeutic targets (Curtin et al., 2015b). While the HERV-W/MSRV Env expression has been documented in both normal and MS brains, making a pivotal role of these proteins in the disease onset unlikely (Grandi and Tramontano, 2017), their increased presence in MS lesions and their ability to trigger adaptive and, especially, innate immunity suggested that they could take part, with other individual factors, to MS immunopathogenesis (Table 3).

**Table 3:** HERV-W/MSRV-derived Env proteins investigated in human autoimmune diseases.

**Disorder**	**Main symptoms**	**Possible Env-related effects**	**References**
Multiple Sclerosis	Progressive demyelination of central nervous system, axon damage, alterations in both innate and adaptive immunity, neuroinflammation	Abundant expression in brain lesions and cells involved in neuroinflammation, superAg activity, potent stimulation of TLR4 and massive production of proinflammatory cytokines responsible for astrocyte and oligodendrocyte damage/death and MS-like disease in mouse models	Perron et al., 2001, 2013; Antony et al., 2004; Rolland et al., 2005, 2006; Mameli et al., 2007b; Saresella et al., 2009; Madeira et al., 2016
		TLR4-mediated induction of nitric oxide synthase, formation of nitrotyrosine groups blocking oligodendrocyte differentiation and affecting myelin expression	Kremer et al., 2013
		Onset of specific humoral response, potential molecular mimicry with myelin protein and Ab cross-reactivity	Brudek et al., 2009; Do Olival et al., 2013; Mameli et al., 2015; Ramasamy et al., 2017
CIDP	Demyelination of peripheral nervous system roots, chronic inflammation	Stimulation of Schwann cells IL-6 and CXCL10 chemokine production, cell-cell fusion, increased cell proliferation, transformation, migration and invasion	Faucard et al., 2016
type 1 diabetes	Immune reactions against pancreatic β cells, deficiency in insulin production	Inhibition of insulin secretion by Langerhans islets β cells, stimulation of pancreatic immune-cell infiltrates	Levet et al., 2017

Regarding innate immunity, syncytin-1 was found to be upregulated in MS patients' brain specific cells involved in neuroinflammation, i.e., astrocytes and microglia, being instead lowly expressed (Antony et al., 2004) or even absent (Mameli et al., 2007a; Perron et al., 2012) in healthy individuals. HERV-W/MSRV Envs were similarly abundant within MS brain lesions, being associated with actively demyelinating sites and expressed principally by macrophages and microglia (van Horssen et al., 2016). A moderate expression was reported in reactive astrocytes within demyelinating areas (van Horssen et al., 2016), and syncytin-1 *in vitro* upregulation prompted the production of proinflammatory molecules that could likely cause astrocyte and oligodendrocyte damage (Antony et al., 2011). In addition, HERV-W/MSRV Env was shown to act like a superAg (Emmer et al., 2014), stimulating the polyclonal T-cell activation and eliciting an abnormal innate response with massive release of multiple cytokines, already known to play a central role in demyelination (Perron et al., 2001; Rolland et al., 2005) (Table 3). Further studies revealed that HERV-W/MSRV Env pro-inflammatory properties depend on the stimulation of toll-like receptor 4 (TLR4) (Perron et al., 2001; Rolland et al., 2006; Saresella et al., 2009), evoking the same proinflammatory cytokines prevalent in MS, such as interleukins (IL-1, IL-6) and tumor necrosis factor α (Rolland et al., 2005, 2006; Mameli et al., 2007b). Such TLR4 activation by Env proteins led also to the induction of nitric oxide synthase, with the formation of nitrotyrosine groups blocking oligodendrocyte differentiation and hence affecting myelin expression and renewal (Kremer et al., 2013) (Table 3). The marked ability of HERV-W/MSRV Envs in triggering innate immunity has been further characterized through murine models (Table 3). The intraperitoneal injection of MSRV virions in humanized immunodeficient mice led to acute neurological inflammation and animal death due to massive brain hemorrhage (Firouzi et al., 2003). A similar outcome was obtained with syncytin-1 overexpression, inducing inflammation, neurobehavioral abnormalities and oligodendrocytes and myelin injuries mediated by redox reactants including nitric oxide (Antony et al., 2004, 2007). Finally, MSRV-Env was confirmed to strongly trigger the TLR4-mediated production of proinflammatory cytokines, leading to experimental allergic encephalomyelitis in mice (Perron et al., 2013). Accordingly, MSRV Env was confirmed to be a potent agonist of TLR4 (Madeira et al., 2016) and a monoclonal Ab neutralizing the protein (GNbAC1) was shown to reduce such immune activation, rescuing myelin expression (Kremer et al., 2014).

Considering adaptive immunity, HERV-W/MRSV Env epitopes have been detected on active MS patient B cells and monocytes (Brudek et al., 2009) and showed potential cross-reactivity against myelin proteins (Ramasamy et al., 2017), being possibly involved in molecular mimicry events. Accordingly, MSRV Env stimulated IFN-γ release by T cells when associated with the myelin oligodendrocyte glycoprotein (MOG) Ag (Perron et al., 2013), and the Env protein encoded by an HERV-W processed pseudogene (ERVWE2, locus Xq22.3) was shown to have five domains similar to the Ig-like domain of MOG, including T and B cell epitopes (Do Olival et al., 2013) (Table 3). In addition, IgG Abs in MS patients were shown to strongly recognize two HERV-W Env peptides, having a slight decline after IFN-β treatment (Mameli et al., 2015). A similar decrease in anti-HERV-W Env Abs following IFN-β therapy was previously reported, albeit being not statistically significant (Petersen et al., 2009). In contrast, another study assessing the humoral response against HERV-W/MSRV found no support for its specificity to MS, detecting syncytin-1 Abs in only 1/50 patients and MSRV Env Abs in none of them (Ruprecht et al., 2008a).

Overall, to date, the sole presence of HERV-W/MSRV Env Ags and Abs in MS patients does not support their association with MS etiology. Contrarily, the evident Env immunopathogenic properties, especially in evoking innate immunity, strongly suggest a role in MS clinical manifestations (Grandi and Tramontano, 2017). However, the fact that HERV-W/MSRV Envs have been reported in healthy controls too likely suggests that their expression represents a physiological phenomenon, possibly showing higher prevalence and pathological consequences in MS due to the altered epigenetic and immunological environment and the presence of other individual triggers (Hon et al., 2013; Sun et al., 2016). Given this multifactorial interplay, involving genetic predisposition and exogenous agents (Christensen, 2005; Ryan, 2011), the HERV-W/MSRV Env superAg activity is the most probable HERV-derived contributor to MS clinical manifestations, inducing inflammatory effects coincident with the major hallmarks of MS (van Horssen et al., 2016). Such possible participation in MS neuropathogenesis makes HERV-W/MSRV Envs promising targets for innovative therapeutic approaches. In this regard, GNbAC1 monoclonal Ab selectively recognizing HERV-W/MSRV Env showed promising neutralizing effects *in vitro* and in mouse models, and is currently under clinical trial (Curtin et al., 2015a,b) (see below).

HERV-W expression has been investigated in CIDP, another autoimmune disease affecting the peripheral nervous system with inflammatory and demyelinating lesions in nerve roots (Faucard et al., 2016). The expression of MSRV-Env was detected in 5 out of 8 CIDP patients (Perron et al., 2012), and the protein was found in the nerve lesions of 5 out of 7 patients (Faucard et al., 2016) with a prevailing expression in Schwan cells (Faucard et al., 2016). The latter exposition to MSRV-Env led to the stimulation of IL-6 and chemokine CXCL10 that was significantly inhibited by GNbAC1 Ab (Faucard et al., 2016).

#### Diabetes

HERV-W Env expression has been recently studied in type 1 diabetes, showing significant upregulation in 70% of diabetes patients as compared to a 12% positivity in healthy controls (Levet et al., 2017). The immunostaining of the protein in pancreatic specimens from 20 cases and 19 controls gave comparable positivity percentages (75 and 16%, respectively), showing a predominant localization in acinar cells, proximal to Langerhans islets (Levet et al., 2017). Furthermore, mice transgenic for HERV-W Env expression developed hyperglycemia, diminished insulin levels and pancreatic infiltrates of immune cells, all hallmarks of type I diabetes (Levet et al., 2017). In particular, the inhibition of insulin secretion was determined by HERV-W Env protein in a dose-dependent manner, being restored in the presence of neutralizing Abs and possibly depending on the protein interaction with pancreatic β cell TLR4 (Levet et al., 2017). Hence, authors suggested that HERV-W-Env might exert a double pathological effect, impairing pancreatic β cell insulin secretion and stimulating autoimmune reactions. It is worth noting that a phase-IIa clinical trial is currently testing GNbAC1 monoclonal Ab as possible HERV-based therapeutic approach in type 1 diabetes (Levet et al., 2017).

### HERV-K

As seen for cancer, the majority of studies tentatively linking HERV-K supergroup to autoimmunity was dedicated to HML2 group, with particular attention to some specific proviruses: HERV-K10 (locus 5q33.3) and HERV-K18 (locus 1q23.3). It is worth noting that members of the same HERV group generally share high identity, and it is thus likely that findings reported for a given element could actually involve related ones too, especially with the use of Abs not characterized for their cross-reactivity with other proteins of the same group. Overall, differently from HERV-W, the involvement of HML2-derived Env in autoimmune diseases is still controversial.

HML2 Envs derived from HERV-K18 provirus were originally proposed to act like superAgs in type 1 diabetes, activating patients' Vβ7 and Vβ13 T lymphocytes and leading to pancreatic β cell damage (Conrad et al., 1997). Later on, such theory has been widely controverted by different studies that reported the absence of both selective expression and immunopathogenic significance of HERV-K18 Env in type 1 diabetes (Badenhoop et al., 1999; Jaeckel et al., 1999; Kim et al., 1999; Muir et al., 1999; Herve et al., 2002) as well as the lack of any superAg activity (Lapatschek et al., 2000).

In rheumatic diseases, two HML2 Env proteins (associated with HERV-K10 and the above mentioned HERV-K18 proviruses) were investigated in systemic lupus erythematosus (SLE) and Sjögren syndrome to assess their ability to stimulate humoral immunity, showing albeit no significant increase of specific Abs as compared to healthy controls (Herve et al., 2002). Similarly, a recent study investigating HERV-K Env humoral recognition in rheumatoid arthritis reported the highest reactivity against a SU epitope that was however recognized at low prevalence by both patients and healthy controls (19 and 3%, respectively) and did not show significant correlation with the disease (Mameli et al., 2017).

### Other HERV groups

In addition to HERV-W and HERV-K, a few studies reported the expression of other HERV Envs and/or the presence of specific Abs in some autoimmune disorders.

Psoriatic and atopic skin samples were generally positive for HERV-E Env as compared to a low positivity in normal skin, being also expressed in CD4+ T cells found in psoriatic lesions (Bessis et al., 2004). Such protein expression, contrary to what observed for HERV-K in melanoma, was downregulated by UV irradiation (Bessis et al., 2004).

Elevated Ab titers against ERV-3 Env were found in healthy pregnant women (accordingly to its known placental expression) and in women affected by either SLE or Sjögren syndrome (Li et al., 1996). Authors reported that, in healthy women, the highest Ab levels were observed in mothers of babies suffering from congenital heart block. This and the evidence of ERV-3 Env expression in both placenta and fetal heart supported the theory of a possible autoimmunization during pregnancy (Li et al., 1996). However, the fact that patients suffering from SLE showed reactivity against HIV-1 and HTLV-1 Envs too could suggest the occurrence of molecular mimicry events (Balada et al., 2010).

### Evidence of HERV-env immunosuppressive activity in autoimmunity

While the majority of studies attempted to find a link between HERV Env and autoimmune reactions, a limited number of works presented instead an opposite scenario, in which these proteins could even downregulate immune activation through their immunosuppressive activity.

In a small group of psoriatic patients, the expression of HERV-K(HML2) *env* was decreased as compared with healthy individuals, with a concomitant decline in the specific Ab response (Gupta et al., 2014). An HERV-H Env protein was upregulated in SLE individuals, being but negatively correlated to IL-6 levels and able to affect the latter production *ex vivo* (Laska et al., 2017). Intriguingly, based on this inverse relation between Env expression and patients immune response, authors proposed that in some instances HERV Envs might have been exploited by human immunity to support a negative inflammatory feedback (Laska et al., 2017). In line with this hypothesis, the upregulation of HERV Env expression, as frequently observed in pathological conditions (e.g., exogenous infections and inflammation), could provide an immunosuppressive system to control harmful cytokine production (Laska et al., 2017). Even if limited, these findings might indicate that Env expression in autoimmunity could constitute a multifaceted phenomenon, arose due to the altered immune and epigenetic conditions and having some relevance for the disease pathogenesis, likely depending on the genetic and environmental background. In addition, the impact of HERV Env expression on immunopathogenesis may be ambivalent, having negative or positive effects based on the prevalence of either immune-stimulatory or immune-suppressive activities. In this view, HERV Envs in healthy population can theoretically serve as a sort of “immune sentinels” that, on the one hand, provide physiological functions and protect the tissues from exaggerate immune reactions and, on the other hand, maintain a basal immune alert leading, in some complex conditions, to harmful effects.

## Toward HERV-based therapies: needs and potential

In line with their proposed role in cancer and autoimmunity, HERVs are considered promising targets for the development of innovative therapeutic strategies. It is however noteworthy that, paradoxically, while the first HERV-based therapies are currently in clinical studies, no human illness has been definitively associated with any HERV, yet. Thus, in general, an important gap that current studies are trying to fill is to provide the definitive evidence of a causal association between HERVs' presence/expression and the onset/progression of a given disorder. Such final demonstration should satisfy the criteria commonly used to assess cause-effect relationships and applied to viral-related pathogenesis as well, relying on the growing amount of *in vitro* and *in silico* screening tools and biostatistics models (Ronit and Shou-Jiang, 2011; Fedak et al., 2015). First of all, even if a weak association does not necessary indicate the absence of causality between the exposure to a given factor and pathogenesis, the stronger is an association, the more likely is its causality. Hence, the presence of HERV proteins in diseased conditions should represent a starting point for evaluating the significant upregulation of specific HERV loci in diseased vs. healthy tissues through appropriate statistical models. Another important point is the consistence, i.e., the reproducibility of observations made in variable conditions (different groups, places, samples, etc.). This is a major goal for HERV studies, because the absence of standardized methodologies and the poor genomic characterization of individual HERV groups led often to discordant results about their expression in human diseases. This unfortunately affected the coherence of epidemiologic literature in the field, with different studies reporting conflicting and poorly comparable associations. Thus, advanced mechanistic analyses and standardized specific methodologies can elucidate which are the most consistent evidence, thereby rationally directing the causal investigation. Moreover, epidemiologic associations should not only support biological hypothesis, but should be plausible according to the existing knowledge about diseases pathogenesis. Hence, besides being expressed in diseased tissues, HERV proteins should hold biological activities potentially explaining the disease molecular and/or clinical manifestations. Accordingly, it is important to explore the interaction with cellular or exogenous partners already involved in the disease pathogenesis, thus considering that many of the accounted diseases arise from the complex interplay between multiple factors. This implies that the manipulation of a sole determinant may produce undetectable or misleading effects, asking for exhaustive and carefully designed experimental systems supported by appropriate animal models. The latter should be used to assess the specificity of HERV expression, elucidating the exact molecular mechanism of pathogenesis through multiple integrated research approaches and considering eventual influencing factors (epigenetics, drug treatments, physical agents, etc.). Similarly, in the case of a complex temporality between exposition and pathogenesis, the presence of HERV products should direct the investigation to specific loci, demonstrating their ability to induce *de novo* the observed effects and considering that HERV expression varies in the different periods of life (e.g., development, pregnancy) or according to epigenetic changes (e.g., cancer, autoimmune diseases). Besides temporality, the biological gradient should be evaluated, given that many HERVs are expressed in both patients and healthy individuals, without apparent harmful effects in the latter. Hence, it would be useful to understand if this could depend on a threshold under which HERV products have not significant impact on the host, being “symptomatic” only when upregulated (even as an epiphenomenon) in diseased contexts. This could help the rational design of protocols testing candidate molecules for HERV-inhibition. Finally, due to the principle of analogy, HERV products belonging to a same group share significant similarity. Hence, if on the particular one side it is unlikely that their expression could indiscriminately produce pathogenesis, asking for more specific procedures, on the other side, the reliable characterization of HERV pathogenic determinants in a given disease could allow to reach lowered standards of evidence for subsequent association studies.

### Development of HERV-based anticancer approaches

Since HERV Envs have been suggested to exert pro-oncogenic effects in a number of tissues, being possibly involved in tumor progression and in the downstream metastatic spread, they constitute promising targets for innovative anti-cancer strategies based either on HERV inhibitors or immunotherapy approaches. While the former clearly requires a supported causative association between HERV expression and cancer progression, the latter can exploit both the selective or upregulated expression of HERV Ags to direct therapeutic agents against cancer cells. To date, various HERV-based anticancer approaches have been proposed, even if, to the best of our knowledge, none of them is currently under clinical development (sources: *clinicaltrials.gov*, USA; *clinicaltrialsregister.eu*, EU).

#### HERV inhibitors

In the presence of a demonstrated role of HERV proteins in cancer onset and/or progression, a valuable therapeutic strategy could be based on molecules or small RNAs inhibiting either the protein activity or the upstream HERV expression. This approach includes the possibility to test the cross-efficacy of antiretroviral molecules already approved for exogenous retroviral and non-retroviral infection treatment. As an example, colorectal cancer cells with an induced chemotherapy-resistant phenotype were shown to hyperexpress HERV-W and HERV-FRD Envs, and such expression was efficiently downregulated by different antiviral compounds (amantadine, ribavirin and pleconaril) (Díaz-Carballo et al., 2015). Interestingly, the combination of antitumor agents and these antiviral drugs led to synergistic antiproliferative effects, increasing the cytotoxicity against the multiresistant colorectal tumor cells (Díaz-Carballo et al., 2015). Similarly, the treatment of prostate cancer cell lines harboring endogenous RT activity with the nucleoside HIV-1 RT inhibitor Abacavir showed marked anti-proliferative effects, even if no data are available about the specific action on HERV expression (Carlini et al., 2010). Also non-nucleoside HIV-1 RT inhibitors Nevirapine and Efavirenz were tested against HERV-K(HML2) expression, reducing proliferation and promoting apoptosis in melanoma cells with induced stemness features (Argaw-Denboba et al., 2017).

#### Passive and active immunotherapy

HERV Envs being upregulated and/or found exclusively in tumor tissues could be suitable targets to direct both passive and active immunotherapy against cancer cells, even in the absence of a direct role in the disease onset and progression.

Passive immunotherapy is mainly based on the development of Abs recognizing the HERV Envs expressed in diseased tissues. Given the high similarity shared by HERV proteins, especially among related groups, the design of selective Abs cannot ignore the need of a proteomic project to characterize the specific expression of individual HERV peptides. Given the high expression of HERV-K(HML2) in hBC, a monoclonal Ab against HML2 Env was shown to inhibit hBC cell line proliferation, with the concomitant activation of apoptotic signals (Wang-Johanning et al., 2012). The same Ab significantly reduced the growth of xenograft tumors in mice, being therefore proposed as possible immunotherapeutic agents for hBC (Wang-Johanning et al., 2012).

While passive immunotherapy relies on Abs administration, active immunotherapy aims to stimulate an intrinsic cellular and humoral response against diseased cells. In particular, an ideal anticancer therapeutic agent should be as selective as possible toward transformed cells only, and should be able to prevent recurrences by evoking a protective immunity (Mullins and Linnebacher, 2012b). Due to its specificity and durability, active immunotherapy is considered more advantageous with respect to passive immunization, even if both approaches might be combined to gain a higher anticancer effect. Currently, various HERV-derived Envs have been investigated for anticancer immunotherapy, being expressed to higher extents (tumor-associated Ags, TAAgs) or exclusively (tumor-specific Ags, TSAgs) in transformed cells. In this context, an important therapeutic opportunity would be the identification of HERV TSAgs shared between different tumors, to develop broad-spectrum anticancer strategies. The first attempt to exploit endogenous retroviral proteins as TAAgs was performed in murine colorectal carcinoma and melanoma cell lines producing ERV Envs, in which recombinant vaccinia virus was used for anti-tumor immunization against these proteins (Yang and Perry-Lalley, 2000; Kershaw et al., 2001). In a similar way, recombinant vaccinia virus expressing HERV-K(HML2) Env reduced the number of nodules of Env-expressing pulmonary tumors induced in mice, which were even prevented by the vaccine prophylactic administration (Kraus et al., 2013). In humans, a multicentric study reported that the incidence of melanoma is reduced in individuals that have received vaccinia and/or bacille Calmette-Guerin vaccination, used to induce protective immunity against smallpox and tuberculosis, respectively (Krone et al., 2005). Such lower melanoma risk was also confirmed in individuals having suffered from acute infectious diseases, possibly suggesting that different viral Ags sharing sequence homologies with HERV-K-MEL-Ags could induce a cross-protection against melanoma development (Krone et al., 2005). An analogous effect was reported in a case report about a patient with metastatic melanoma who achieved spontaneous cancer regression after a febrile reaction to tetanus–diphtheria–pertussis combined vaccination (Tran et al., 2013). Likewise, given the antigenic similarity between HERV-K-MEL and yellow fever virus (YFV) (Krone et al., 2005), cohorts of individuals having received anti-YFV vaccination were investigated for melanoma incidence, showing however no significant protective effects in the 10 years post-vaccination (Mastrangelo et al., 2009; Hodges-Vazqueza et al., 2012). In addition to the findings in melanoma cells (Schiavetti et al., 2002), HERV-K-MEL expression and possible exploitation for immunotherapeutic purposes have been investigated in pancreatic cancer (Schmitz-winnenthal et al., 2007), driving the production of engineered chimeric T cells that lysed tumor cells expressing HERV-K-MEL and decreased tumor mass when injected in a mouse model of metastatic melanoma (Zhou et al., 2015; Krishnamurthy et al., 2016). A subset of gastrointestinal cancers showed significant upregulation of the Env encoded by a HERV-H provirus (locus Xp22.3), and T cells sensitized toward such protein had lytic effects against colorectal carcinoma cells that expressed it (Mullins and Linnebacher, 2012a). Similarly, a HERV-E Env selectively expressed in renal carcinoma was shown to induce cytolytic T lymphocytes recognizing renal carcinoma cells (Haruta et al., 2015).

#### Combination with demethylating agents

Demethylating drugs are commonly used as anticancer agents and are known to liberate retrotransposon expression by inducing a hypomethylated status. Remarkably, the antitumor activity of DNA methyltransferase inhibitors is thought to rely on this trigger toward HERV expression, stimulating the production of viral dsRNA that is sensed as a PAMP by cellular recognition pathways, leading to the immune attack against tumor cells (Roulois et al., 2015). Accordingly, the individual knock-down of MDA5, MAVS and IRF7 PPRs in colorectal cells significantly reduced the anticancer activity of DNA methyltransferase inhibitors (Roulois et al., 2015). Therefore, considering that the sole immune checkpoint therapy often produces weak responses in cancer patients, demethylating agents have been proposed in combination with active immunization to produce synergistic anticancer effects (Chiappinelli et al., 2016).

### HERV-based therapeutic treatments in autoimmunity

HERV Envs showed remarkable immunogenic properties suggesting their contribution to autoimmune diseases through both molecular mimicry and superAg activities (Emmer et al., 2014; Trela et al., 2016). They were hence investigated as possible targets for innovative autoimmunity therapies, mainly focused on either the inhibition of HERV expression or the passive immunization against HERV Env proteins. To date, at least three clinical trials are dedicated to the development of HERV-Env based therapies for MS (2) and type I diabetes (1) (sources: *clinicaltrials.gov*, USA; *clinicaltrialsregister.eu*, EU).

#### HERV inhibitors

As reported for cancer, in the presence of a pathological contribution of HERV products, the use of molecules inhibiting such proteins' activity or expression could reduce the associated clinical manifestations. Intriguingly, it has been hypothesized that the cytoplasmic accumulation of endogenous retroelements could led to the activation of innate DNA sensors and the consequent production of IFN in mice, especially in the presence of mutations affecting the 3′ repair exonuclease 1 (Trex1) (Stetson et al., 2008; Gall et al., 2012; Xu et al., 2014). Even if the actual role of human Trex1 in the degradation of cytosolic HERV cDNA is still to be demonstrated, it has been shown that this exonuclease can metabolize reverse-transcribed endogenous retroelements, which accumulates on the contrary in the cytosol of mouse Trex1-deficient cells (Stetson et al., 2008). Accordingly, mice affected by hereditary autoimmune inflammation and deficient for Trex1 were treated with HIV-1 RT inhibitors showing a significant amelioration in symptomatology (Beck-Engeser et al., 2011). Similarly, a case report described that the administration of Raltegravir HIV-1 IN inhibitor led to the stable remission of a severe autoimmune chronic idiopathic urticarial patient that showed resistance to all traditional treatments (Dreyfus, 2011). Thus, the administration of HERV inhibitors could be theoretically suitable to treat some immune manifestations linked to HERV replication products, although further studies are needed to understand the significance of the latter accumulation in autoimmunity and the cellular networks involved in their sensing and degradation.

#### Passive immunotherapy

The natural onset of Abs against HERV Envs in autoimmunity patients suggested that specific neutralizing Abs could be developed for therapeutic purposes. Until now, the main findings regard the above-mentioned GNbAC1 monoclonal Ab, targeting HERV-W/MSRV Env proteins and proposed as innovative therapy for MS and type I diabetes. In fact, GNbAC1 inhibited the release of proinflammatory cytokines by PBMC stimulated with HERV-W Env (Rolland et al., 2006) and reduced the HERV-W Env-dependent TLR4-mediated induction of nitric oxide synthase, rescuing myelin expression and oligodendrocyte differentiation (Kremer et al., 2014). The Ab was then tested in an HERV-W Env-induced experimental allergic encephalitis mouse model (Perron et al., 2013), corroborating its efficacy in ameliorating the disease symptoms and preventing the animals death (Curtin et al., 2015b). The early clinical development assessed favorable safety and pharmacokinetic profiles (Derfuss et al., 2015b; Curtin et al., 2016) that were confirmed in a phase IIa randomized study of 10 MS patients, showing positive pharmacodynamics responses (Derfuss et al., 2015a,b). Aside from MS, a pathological role of HERV-W Env has been suggested in insulin deficiency and type I diabetes immunopathogenesis (Levet et al., 2017). Subsequently, GNbAC1 Ab is currently tested in a phase-IIa clinical study of type I diabetes patients, being possibly an important clinical option in treating this autoimmune disease (Levet et al., 2017).

## Conclusions

HERV-derived Env proteins constitute multifaceted and multifunctional elements at the interface between self and non-self, showing a delicate balance of the same biological activities in serving the host physiology and exerting harmful effects. Our understanding of HERVs has grown in the last three decades and, by now, it is evident that their expression is a normal phenomenon and, thus, cannot be used as the only evidence of their involvement in human disorders. It is hence necessary to characterize individual HERV proteins for their actual effects on the molecular pathways involved in human pathogenesis, to finally individuate precise causalities and allow the effective exploitation of selected elements as specific biomarkers and promising therapeutic targets.

## Author contributions

NG and ET participated to the conception, drafting and revision of the manuscript and approved the final version.

### Conflict of interest statement

The authors declare that the research was conducted in the absence of any commercial or financial relationships that could be construed as a potential conflict of interest.
